# The effects of different types of organisational workplace mental health interventions on mental health and wellbeing in healthcare workers: a systematic review

**DOI:** 10.1007/s00420-024-02065-z

**Published:** 2024-05-02

**Authors:** Birgit Aust, Caleb Leduc, Johanna Cresswell-Smith, Clíodhna O’Brien, Reiner Rugulies, Mallorie Leduc, Doireann Ni Dhalaigh, Arilda Dushaj, Naim Fanaj, Daniel Guinart, Margaret Maxwell, Hanna Reich, Victoria Ross, Anvar Sadath, Katharina Schnitzspahn, Mónika Ditta Tóth, Chantal van Audenhove, Jaap van Weeghel, Kristian Wahlbeck, Ella Arensman, Birgit A. Greiner, MENTUPP consortium members, Ainslie O’Connor, Ana Moreno-Alcázar, Andia Meksi, Andras Szekely, Anthony LaMontagne, Ariel Como, Arlinda Cerga Pashoja, Asmae Doukani, Azucena Justicia, Benedikt Amann, Bridget Hogg, Charlotte Paterson, Chris Lockwood, David McDaid, Eva Zsak, Eve Griffin, Evelien Coppens, Fotini Tsantila, Genc Burazeri, Gentiana Qirjako, György Purebl, Ilinca Serbanescu, Jeroen Luyten, Joe Eustace, Joseph Kilroy, Juan Carlos Medina Alcaraz, Juliane Hug, Kairi Kõlves, Kahar Abdulla, Karen Michell, Karen Mulcahy, Katherine Thomson, Lars de Winter, Laura Cox, Lia van der Ham, Luigia D’Alessandro, Marta Fontana, Nicola Reavley, Peter Trembeczky, Pia Driessen, Pia Hauck, Paul Corcoran, Rebecca Lohmann-Devantier, Saara Rapeli, Sarah Ihinonvien, Sevim Mustafa, Stefan Hackel, Susan Alexander, Tanya King, Ulrich Hegerl, Vanda Scott, Wendy Orchard

**Affiliations:** 1 National Research Centre for the Working Environment, Copenhagen, Denmark; 2 School of Public Health, University College Cork, Cork, Ireland; 3 Finnish Institute for Health and Welfare, Helsinki, Finland; 4 National Suicide Research Foundation, University College Cork, Cork, Ireland; 5 Section of Epidemiology, Department of Public Health, University of Copenhagen, Copenhagen, Denmark; 6 Community Centre for Health and Wellbeing, Tirana, Albania; 7 Per Mendje Te Shendoshe (PMSH), Prizren, Kosovo; 8 Alma Mater Europaea Campus Rezonanca, Pristina, Kosovo; 9 CIBERSAM, Hospital del Mar Research Institute, Barcelona, Spain; 10 Institut de Salut Mental, Hospital del Mar, Barcelona, Spain; 11 The Donald and Barbara Zucker School of Medicine at Hofstra/Northwell, Hempstead, USA; 12 Nursing, Midwifery and Allied Health Professionals Research Unit, University of Stirling, Stirling, Scotland; 13 German Foundation for Depression and Suicide Prevention, Leipzig, Germany; 14 Depression Research Centre of the German Depression Foundation, Department of Psychiatry, Psychosomatic Medicine and Psychotherapy, University Hospital, Goethe University, Frankfurt, Germany; 15 Australian Institute for Suicide Research and Prevention, WHO Collaborating Centre for Research and Training in Suicide Prevention, School of Applied Psychology, Griffith University, Brisbane, Australia; 16 European Alliance Against Depression, Frankfurt, Germany; 17 Institute of Behavioural Sciences, Semmelweis University, Budapest, Hungary; 18 KU Leuven, Louvain, Belgium; 19 Center for Care Research and Consultancy, LUCAS, Louvain, Belgium; 20 Tranzo Scientific Center for Care and Wellbeing, Tilburg University, Tilburg, The Netherlands

**Keywords:** Burnout, Job stress intervention, Workplace mental health intervention, Effectiveness, Small-to-medium size enterprise, Wellbeing

## Abstract

**Objective:**

To determine if and which types of organisational interventions conducted in small and medium size enterprises (SMEs) in healthcare are effective on mental health and wellbeing.

**Methods:**

Following PRISMA guidelines, we searched six scientific databases, assessed the methodological quality of eligible studies using QATQS and grouped them into six organisational intervention types for narrative synthesis. Only controlled studies with at least one follow-up were eligible.

**Results:**

We identified 22 studies (23 articles) mainly conducted in hospitals with 16 studies rated of strong or moderate methodological quality. More than two thirds (68%) of the studies reported improvements in at least one primary outcome (mental wellbeing, burnout, stress, symptoms of depression or anxiety), most consistently in burnout with eleven out of thirteen studies. We found a strong level of evidence for the intervention type “Job and task modifications” and a moderate level of evidence for the types “Flexible work and scheduling” and “Changes in the physical work environment”. For all other types, the level of evidence was insufficient. We found no studies conducted with an independent SME, however five studies with SMEs attached to a larger organisational structure. The effectiveness of workplace mental health interventions in these SMEs was mixed.

**Conclusion:**

Organisational interventions in healthcare workers can be effective in improving mental health, especially in reducing burnout. Intervention types where the change in the work environment constitutes the intervention had the highest level of evidence. More research is needed for SMEs and for healthcare workers other than hospital-based physicians and nurses.

**Supplementary Information:**

The online version contains supplementary material available at 10.1007/s00420-024-02065-z.

## Introduction

Healthcare systems need healthy workers (De Lange et al. 2020). Attention to health care workers general health and especially mental health is therefore crucial and particularly so during a global health crisis like the COVID-19 pandemic (Vizheh et al. 2020; Woo et al. 2020; Chigwedere et al. 2021). Systematic reviews indicate that burnout among physicians and nurses is a widespread phenomenon (Dewa et al. 2014; Woo et al. 2020). Shanafelt et al. (2012) found among physicians, the highest rates of burnout occurring in front-line workers (family medicine, general internal medicine, and emergency medicine) and among nurses, higher prevalence rates for burnout have been found in intensive and critical care and emergency care (Adriaenssens et al. 2015; Woo et al. 2020).

In 2022, the health and social services sector featured prominently in terms of high levels of self-reported work stress, depression and anxiety in Europe (30% compared with 27% for all EU-27 workers), most at risk to the exposure of violence and verbal abuse (30% compared with 16% for all EU-27 workers) and severe time pressure and work overload (51% compared with 46% for all EU-27 workers) (Leclerc et al. 2022). In addition, by the nature of their work, healthcare workers are particularly vulnerable to critical incidents during work, such as dealing with unexpected death or patient violence, which may result in increased risk of post-traumatic stress symptoms, anxiety and depression symptoms (de Boer et al. 2011).

Reduced mental health in healthcare workers can have consequences beyond affected individuals and lead to negative healthcare performance such as reduced quality for patient care, risk of accidents, absenteeism, lower organisational commitment and increased turnover (Salyers et al. 2017; West et al. 2018; Wei et al. 2018; Jun et al. 2021). Mental health in the healthcare sector has therefore gained increased attention also within national healthcare performance enhancement strategies. For example, in the United States, researchers have called for broadening the national triple aim strategy (Bodenheimer and Sinsky 2014) which includes the simultaneous pursuit of improving the patient experience of care, improving the health of populations, and reducing health care costs (Berwick et al. 2008) to a broader quadruple aim strategy where the health of healthcare workers is added as a fourth aim acknowledging that a healthy workforce is of paramount importance in achieving the three original aims (Bodenheimer and Sinsky 2014; Jacobs et al. 2018).

Providing healthy workplaces is also important for the recruitment and retention of healthcare workers (Wallace 2017), especially while the competition for healthcare professionals is increasing due to an aging population in many societies. The World Health Organisation (WHO) has predicted massive shortages of qualified healthcare professionals, in particular nurses (Woo et al. 2020). Knowing more about how to create mentally healthy workplaces can therefore be seen as an important contribution, as it has been found that work-related stress, workforce burnout, and leadership support are factors that influence retention and turnover (Halter et al. 2017; de Vries et al. 2023).

### The psychosocial work environment of healthcare workers and the need for organisational interventions

The important role that good psychosocial working conditions play for workers’ mental health and wellbeing has been pointed out by scholars for decades and synthesised in recent meta-analyses of high-profile epidemiological studies (Theorell et al. 2015; Aronsson et al. 2017; Niedhammer et al. 2021). The exposure to detrimental psychosocial working conditions became particularly evident and more pronounced during the COVID-19 pandemic (Sahebi et al. 2021; Harvey et al. 2021). In addition, ongoing medical developments contribute to growing work demands, speed, complexity, and responsibility for healthcare workers (Adriaenssens et al. 2015) while the administrative burden has been increasing for physicians and work-autonomy decreasing (Harvey et al. 2021). Exposure to physical and verbal violence were documented as very high, especially in nurses and physicians particularly those working in emergency and psychiatric settings (Liu et al. 2019) and in elderly residential care where employees often work alone (OECD 2020). In addition, the increased use of complaints procedures puts extra pressure on physicians with negative impact on physician functioning and mental health (Bourne et al. 2015, 2016). Based on this evidence, researchers for many years have called for more focus on work-directed approaches, also called organisational interventions, than on the widespread worker-directed approaches, also called individual interventions (Semmer 2006; LaMontagne et al. 2007). Organisational interventions address workplace psychosocial factors that can affect mental health and wellbeing of workers and as defined by the recently published WHO guidelines on mental health at work “They are planned actions that directly target working conditions with the aim of preventing deterioration in mental health, physical health, quality of life and work-related outcomes of workers. The interventions can include activities directed at teams.” (World Health Organisation 2022). Typically, interventions include the introduction of flexible working arrangements, worker involvement in decisions about their jobs and modification of workload (World Health Organization and International Labour Organization 2022). While individual (worker-directed) approaches, aim to improve the individual worker’s competencies, knowledge, and strengths to cope with working conditions, organisational (work-directed) approaches, aim to improve working conditions and the organisation of work (Aust et al. 2023; Rugulies et al. 2023). Following the principles of the “hierarchy of controls” for occupational safety and health (Montano et al. 2014; Ajslev et al. 2022), it is argued that it is more effective to reduce or eliminate the risks for health and safety than to mitigate risks through individual protection. With regard to mental health interventions, it is assumed that interventions that aim to improve psychosocial working conditions through organisational interventions will be more effective for preventing mental health difficulties and promoting mental health, than (only) improving coping strategies through individual interventions (LaMontagne et al. 2014).

### Workplace mental health interventions in healthcare

Systematic review results (Joyce et al. 2016) and evidence-based mental health intervention frameworks (Nielsen et al. 2018; Petrie et al. 2018; LaMontagne et al. 2019; De Angelis et al. 2020; Harvey et al. 2021) point toward a coordinated range of different approaches needed for workplace mental health interventions extending from prevention of mental health difficulties, to recovery and return-to-work strategies, addressing both the individual worker and the organisational level. However, most research into workplace mental health interventions targets individual level change, e.g., individual coping strategies and resilience (Kunzler et al. 2020) or mindfulness (Lomas et al. 2018). Research into organisational-level interventions modifying psychosocial working conditions or interventions implemented at the level of supervisors and managers to improve working conditions, are less prevalent (Stansfeld and Candy 2006; Theorell et al. 2015; Aronsson et al. 2017; Harvey et al. 2021; Rugulies et al. 2023). One reason may be that individual-level interventions tend to be easier to implement and to evaluate (LaMontagne et al. 2012; Xu et al. 2020). The popularity of individual-level studies might also be due to effectiveness of organisational interventions in the healthcare sector producing mixed results (Ruotsalainen et al. 2015; West et al. 2016; Panagioti et al. 2017; Dreison et al. 2018; Xu et al. 2020). Several factors may explain these varying results for example the use of different outcomes and including different professional settings for evidence synthesis, lack of control groups and, most notably, the large variety of the organisational interventions. With a few exceptions (DeChant et al. 2019; Fox et al. 2022; Naeeni and Nouhi 2023), most reviews investigated whether organisational interventions work (West et al. 2016; Panagioti et al. 2017; Duhoux et al. 2017; Xu et al. 2020; De Simone et al. 2021), without differentiating between different types of organisational interventions. To date, a comprehensive review about how specific types of organisational interventions differ in their effectiveness on a wider range of mental health outcomes and for all occupational groups in health care is lacking. In addition, little is known about the effectiveness of organisational interventions on mental wellbeing (Gray et al. 2019), defined as a positive component of mental health (Keyes 2005). Integrated workplace based mental health interventions embrace the use of strategies to not only prevent harm to mental health and support those with mental health problems, but also to promote the positive by supporting the strengths and capacities in workers, and building ‘healthy’ workplaces (LaMontagne et al. 2019).

### Small-to-Medium sized Enterprises (SMEs)

While workplace mental health interventions are necessary to promote workers mental health, most research in this area is conducted in larger organisations while research in Small-to-Medium sized Enterprises (SMEs) is limited, despite the fact that in OECD countries, SMEs account for about 99% of businesses and for about 70% of jobs (OECD 2017). Following the definition used by the EU (European Commission 2003), SMEs are defined as enterprises with up to 249 employees. It has been suggested that workplace mental health interventions in SMEs may require specific approaches (Martin and LaMontagne 2018; Martin et al. 2020) as they usually have limited financial, structural and personnel resources to engage in mental health promotion and psychosocial workplace risk assessment compared to larger companies and are commonly less likely to engage in workplace health promotion (McCoy et al. 2014). There is very little evidence synthesis on what types of workplace mental health interventions work in SMEs, particularly in healthcare SMEs (Hogg et al. 2021).

To address this gap, we have conducted this review with a specific focus on SMEs in healthcare e.g. small hospitals and nursing homes, private physical therapy or dental practices. It also supports the knowledge base for the ongoing EU-project Mental Health Promotion and Intervention in Occupational Settings (MENTUPP) of which this review is a part of. MENTUPP focusses on SMEs in three sectors: healthcare, information and communications technology (ICT) and construction (https://www.mentuppproject.eu/publications/). Based on a theory of change (Tsantila et al. 2023), the MENTUPP intervention aims to promote wellbeing, reduce both clinical (depressive, anxiety disorders) and nonclinical (stress, burnout) mental health issues and the stigma of mental (ill-) health (Arensman et al. 2023). A review about organisational level mental health interventions in the construction industry has been published (Greiner et al. 2022) and a publication of a review about these type of interventions in ICT workers is currently in preparation.This systematic review was performed in order to synthesize the effectiveness of organisational interventions on mental health outcomes in healthcare workers. The current approach will classify different organisational interventions, in order to assess the effectiveness of specific types of organisational mental health interventions in workplaces of all sizes and specifically in SMEs. Particular attention will be placed on how effective organisational interventions are in terms of reducing harmful working environments and promoting positive working environments (LaMontagne et al. 2019).

#### Objectives

The specific aims of this review are:

To assess the effectiveness of organisational mental health interventions in reducing stress, burnout, depressive and anxiety symptoms, and promoting mental wellbeing in healthcare workers in organisations of all sizes and (2) to assess the effectiveness of organisational mental health interventions in reducing stress, burnout, depressive and anxiety symptoms and promoting mental wellbeing specifically in SME based health care workers.

## Methods

The protocol for this systematic review has been registered with the International Prospective Register of Systematic Reviews (PROSPERO 2020 CRD42020183648, available at: https://www.crd.york.ac.uk/prospero/display_record.php?ID=CRD42020183648 (Greiner et al. 2020). The systematic review was conducted in accordance with PRISMA 2020 guidelines (Page et al. 2021).

### Search strategy

A comprehensive search strategy was developed to identify primary research investigating the effects of organisational mental health interventions on aspects of mental health and wellbeing including stress, burnout and symptoms of depression and anxiety in healthcare workers. The search was performed using six databases in July 2020 and updated on July 9, 2021: Academic Search Complete, CINAHL, PsycINFO, PubMed, Scopus and Web of Science. We did not update the search further, to not include studies conducted under the extraordinary circumstances during the COVID-19 pandemic. We limited our search to articles from 2010 onwards, because our aim was to assess organizational workplace interventions in the current context. We recognized that working conditions in healthcare are constantly changing (Martin 2002; Beschoner et al. 2020), as we have delineated in more detail in the section “The psychosocial work environment of healthcare workers and the need for organisational interventions” and therefore decided to restrict the literature to publications since 2010. The search strategy was developed according to the PRESS guidelines and in an iterative process using the Population, Intervention, Comparison and Outcome (PICO) framework (McGowan et al. 2016). Developed in consultation with the subject librarian within CL/BG’s institution, the search strategy was composed of free-text and controlled vocabulary for healthcare workers (e.g., physicians, nurses, etc.), intervention type (e.g., workplace mental health promotion), study design (e.g., randomised or cluster randomised controlled trials, etc.) and outcomes (e.g., stress, burnout, mental wellbeing, etc.) linked together using Boolean operators. The search strategy underwent review by a second and independent subject librarian in the lead author’s (BA) institution. The search strategy can be found in appendix 1. Articles published in English and since January 2010 were eligible for review to reflect intervention activities in the context of a modern healthcare environment. Unpublished, ‘grey literature’ was not included. Both backward and forward citation chaining of all included articles and selected systematic reviews was conducted to identify additional studies that may have met the search criteria but not located in the search results.

### Inclusion and exclusion criteria

Studies were included if they reported organisational mental health interventions targeted at workers and/or managers within the healthcare sector. Only studies with a control group were deemed eligible as they provide the most robust evidence. Complete inclusion and exclusion criteria can be found in Table 1. Primary outcomes included quantitative measurements of wellbeing, stress, burnout and symptoms of depression and anxiety, secondary outcomes included absenteeism from work, especially where available linked to mental health or wellbeing issues, and psychosocial work environment changes, e.g., work demands, control and influence, social support by peers and by supervisors or managers measured by validated scales. As organisational interventions target the psychosocial work environment, these changes were deemed important intermediary effects of mental health interventions.

**Table 1:** Inclusion and exclusion criteria

Criteria	Description	Inclusion	Exclusion
Population	Healthcare industry	Employees and managers in healthcare according to the NACE classifications covered in Section Q, Division 86 and 87 for human health activities and residential care activities (EUROSTAT 2008) Full-time or part-time employees	Mainly non-working populations (unemployed, retired, long-term sick leave) Populations not working in healthcare Medical students in placements or internships or other healthcare workers in training Clinical populations with mental health disorders
Intervention	Organisational-level mental health intervention	Organisational-level intervention aimed at improving workers' mental health and/or wellbeing or protecting workers from mental health symptoms or disorders, at the level of the organisation by changing aspects of the psychosocial work environment (e.g., organisational policies, leadership style, workplace culture, working conditions) or through systematic training of work-related competencies Interventions designed to or that involve mental health knowledge and awareness building in the organisation or programs to train managers to initiate workplace changes Multi-level interventions targeting organisational and individual changes	Individual-level interventions solely aimed at changing employees’ individual coping skills or behaviour and not embedded into the organisation Health promotion not primarily targeted at mental but at physical health Mental health interventions not formally implemented in the workplace Interventions that solely target individuals with a defined mental health disorder or disease for treatment and referral Interventions that solely target return-to-work after absenteeism due to mental health difficulties Evaluations focussing exclusively on the economic effects of mental health interventions
Comparison	Control group	All experimental study designs with a comparison group, including RCTs, cRCTs, controlled before- and after- designs and controlled quasi-experimental studies	Uncontrolled pre- and post-intervention comparison designs Observational study designs and study designs with a single measurement Studies using solely qualitative research methods
Outcomes	Primary: Mental health and wellbeing	Non-clinical mental health outcomes including stress, burnout, non-clinical depression and anxiety symptoms, mental wellbeing Measured by validated scales or validated physiological indicators	Clinical mental health outcomes: depression and anxiety disorders, other mental health disorders, substance abuse disorder. Suicide related outcomes including suicidal ideation
	Secondary: organisational	Absenteeism Psychosocial work environment changes, including, but not limited to work demands, control/influence, social support by peers and by supervisors/managers measured by validated scales	Presenteeism, turnover intention, productivity, job satisfaction, culture, stigma or work engagement

### Study identification

One researcher conducted the search in the respective databases (CL or CO’B). Results were exported into Rayyan QCRI (Ouzzani et al. 2016), a software application to facilitate study selection in systematic reviews. Duplicates were eliminated with the use of the Rayyan duplicate detection feature and verified by one reviewer (CL). To ensure adequate understanding and consistency in application of the inclusion and exclusion criteria, a sample of 25 records were selected at random and their titles and abstracts were reviewed independently by five authors (BA, CL, CO’B, JC-S, BG). The five authors then met to discuss their inclusion decisions and any discrepancies were discussed until unanimous agreement was reached. Subsequently, 25% of the remaining records were randomly selected and reviewed blindly at the title and abstract level for inclusion by two reviewers (CL and CO’B). Agreement between the reviewers was 99.4% with the discrepancies resolved through discussion and did not require the input of a third reviewer. The remaining 75% of records were then screened at the title and abstract level by one reviewer (CL or CO’B). Blind screening of full-text articles was completed by two authors (CL and BA), who agreed on final inclusion and exclusion decisions.

### Data extraction

Data extraction for the articles after full-text review was completed by one reviewer (CL) included the following and was independently cross-checked by a second reviewer (CO’B): (1) Author and year; (2) type of study design; (3) number of participants and demographics, including employment type; (4) number of control participants and demographics (initial and analysed); (5) intervention details; (6) number of sessions and length; (7) type of control; (8) length of follow-up; (9) relevant outcomes; (10) instruments applied to measure outcomes; (11) country; (12) mean and standard deviation of all study groups in the relevant outcomes at all assessment times to be analysed; and (13) size of the organisation(s). Where data were missing, incomplete or unclear, other sources of information, such as a corresponding protocol article or research report were consulted or requests for additional information were sent to the corresponding study authors by email.

### Quality appraisal

Six areas of methodological quality of each included study were appraised using the “Quality Assessment Tool for Quantitative Studies” (QATQS) scale (McMaster University 2010): (1) selection bias, (2) design, (3) confounders, (4) blinding, (5) data collection method, and (6) withdrawals and drop-outs. Results were scored on a scale from 1 to 3, where 1 is considered methodologically strong, 2 moderate and 3 weak and then globally ranked as methodologically ‘strong’ = no weak ranking, as ‘moderate’ = one weak ranking, and as ‘weak’ with two or more weak rankings. All studies were blindly appraised for quality using the QATQS by two independent reviewers (CL and CO’B or BA and BG) and any disagreements were discussed between the reviewers and resolved.

### Data presentation and synthesis

We conducted a narrative synthesis of the findings regarding the effectiveness of the mental health intervention programmes for primary and secondary outcomes. The synthesis was guided by the aim to identify which types of interventions were effective or not effective for the primary outcomes. To this purpose we built upon a classification developed in a systematic review on the effectiveness of organisational wellbeing interventions by Fox and colleagues (2022). Data were also synthesised by company-size following our objective to evaluate which type of interventions particularly work in SMEs. As many smaller healthcare organisation are part of a larger organisation or public health system, we made the following distinction: We classified the included studies in four main categories according to the intervention organisations (1) large organisation(s) = 250 employees and above, (2) independent SME(s) = below 250 employees and not part of a larger organisation, (3) SME(s) as part of a larger organisation, (4) varied = mixed sample including SMEs. In the context of health promotion, this distinction was deemed to be relevant, as smaller organisations often do not have the capacity to offer health promotion whereas if they belong to a larger umbrella organisation they may have access to their health promotion support systems.

Mean differences (pre- and post- intervention) or adjusted regression coefficients with p-values and with 95% confidence intervals, if available, were tabulated by intervention type and summarized using narrative synthesis. Reported outcomes, that were not specified as primary or secondary outcomes for the purpose of this review were not included in the synthesis. It was not possible to conduct a meta-analysis for the included studies due to the diversity of outcomes and outcome measurements.

We rated the certainty of evidence for each of the six intervention types using an adapted version of a rating system, developed by Heijkants et al. (2023) that allows summarising the level of evidence across studies with heterogenous outcome measures within an overarching concept. The 3-step approach included: (1) Statistically significant impact of the intervention type: 50% or more of the measured primary outcomes (wellbeing, stress, burnout, symptoms of depression or symptoms of anxiety) were significantly improved; (2) Consistency of results for the intervention type: 75% or more of the studies that measured at least one of the 5 primary outcomes (wellbeing, stress, burnout, symptoms of depression or symptoms of anxiety) showed a statistically significant impact in the same direction) and; (3) Summary rating of the level of evidence with consideration of (1) and (2) and of the studies’ QATQS global score resulting in one of the four levels of evidence ratings: 'strong’ (consistent findings in multiple high-quality studies), ‘moderate’ (consistent findings in one high-quality and/or multiple moderate-quality studies), ‘weak’ (consistent findings in one moderate-quality and/or multiple low-quality studies), or ‘insufficient’ (only one study available or inconsistent or null findings in multiple studies).

## Results

### Included studies and study characteristics

**Fig. 1:**
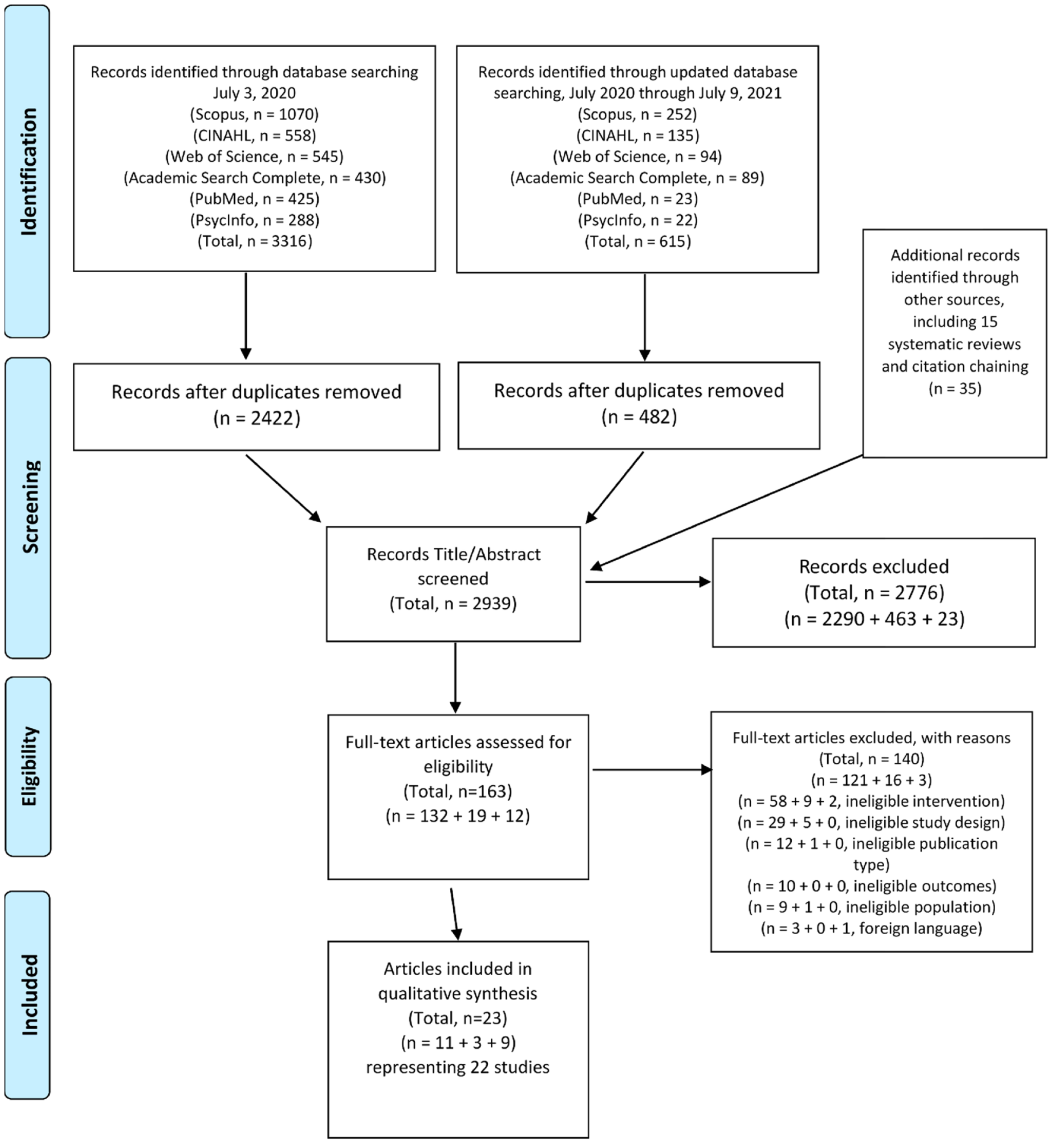
PRISMA flow diagram

At first search, 3316 records were identified from six databases, with an additional 615 records identified as the search was updated through July 9, 2021. A further 35 records were identified through citation chaining of included records, and by a manual review of the included studies and of 15 additionally identified systematic reviews. Following removal of duplicates, the title and abstracts of 2939 records were reviewed for eligibility. After 2776 records were excluded as ineligible, the full text of 163 articles was reviewed. Primary reasons for exclusion at full text review were ineligible interventions (N = 69) and ineligible study designs (N = 34). After applying all inclusion and exclusion criteria, 23 articles relating to 22 studies were identified as eligible for inclusion in the review (see Fig. 1: PRISMA flow diagram).

The 23 identified articles based on 22 studies were published between 2010 and 2021 and included an overall sample of N = 6303 healthcare workers. Table 2 shows the main characteristics of the included studies. Twenty studies were conducted in high-income countries with the bulk of research stemming from the United States (seven studies) and Canada (three studies), while two studies each came from Australia, United Kingdom, The Netherlands and Iran and one study each from Belgium, Denmark, Spain and Japan. Nine studies were carried out with nurses and/or nursing attendants only, six studies were conducted among a mix of job groups, five studies targeted physicians only, one study included homecare workers only, and one study included eldercare workers only. Fourteen studies were conducted within a hospital setting, three studies were conducted in primary care centres, two studies in long-term care units, and one study each in home-based care, a health trust and a health service facility for mental health patients. Half of the studies (11 studies) were conducted in large organisations. None of the studies were conducted in independent SMEs, but five studies were conducted in SMEs that were part of a larger organisational structure.

**Table 2:** Study and sample characteristics

First author, year	Country	Type of study/design	Job group	Number and Size of organisations*	Total sample size (Gender)	Intervention group sample size (Gender)	Control group sample size (Gender)
Ali et al. (2011)	United States	Cluster randomised control trial	Physicians in ICU units	4 Size: Not reported (Assumed to be large: ICUs in four academic hospitals)	39 at baseline (24% female,76% male)	14 worked under the interrupted schedule only (intervention), 13 worked under both types of schedules (Gender not reported)	12 worked under the continuous schedule only, 13 worked under both (Gender not reported)
Barcons et al. (2019)	Spain	Cohort analytic (two group pre + post)	General Practitioners	4 primary care services Size: SMEs as a part of a larger organization Subjects had to be GPs working in public primary-care units. These units also had to belong to the assistance sector of the ambulatory mental-health service	38 at baseline (Gender not reported)	20 (Gender not reported)	18 (Gender not reported)
Bourbonnais et al. (2011)	Canada	Cohort analytic (two group pre + post)	Nurses and attendants	2 Size: Large	1110 at baseline (77.7% female, 22.3% male)	492 (75% female, 25% male)	618 (80% female, 20% male)
Cordoza et al. (2018)	United States	Randomised cross over design	Nurses and nurse managers	1 Size: Large	29 at baseline (93% female, 7% male)	29 (93% female, 7% male)	Participants served as their own controls
Deneckere et al. (2013)	Belgium	Post-test only Cluster Randomised control trial	Medical Doctors, Head Nurses, Nurses, and Allied Health Professionals	23 Size: Large	581 at baseline (Gender not reported)	346 (Gender not reported)	235 (Gender not reported)
Emani et al. (2020)	Iran	Randomised control trial	Nurses in ICU	1 Size: Large	80 at baseline (Gender not reported)	40 (Gender not reported)	40 (Gender not reported)
Garland et al. (2012)	Canada	Cross over design	Internists, Anesthesiologists, and Surgeons in ICU units	2 Size: Large	37 at baseline (8% female, 92% male)	24 (Gender not reported)	Participants served as their own controls
Gregory et al. (2018)	United States	Cohort analytic (two group pre + post)	Primary care physicians	8 clinic sites Size: Not reported	69 at baseline (Gender not reported)	37 (Gender not reported)	32 (Gender not reported)
Havermans et al. (2018)	The Netherlands	Cluster-controlled study	Teams of employees within a large healthcare organisation that facilitates care in nursing homes (including geriatric rehabilitation care), residential care homes, and home-based care	1 Size: Large	304 at baseline (96.7% female, 3.3% male)	161 (95% female, 5% male)	143 (99% female, 1% male)
Jakobsen et al. (2017)	Denmark	Cluster randomised control trial	Female Healthcare Workers	3 Size: Large	200 at baseline (100% female)	111 (100% female)	89 (100% female)
Kossek et al. (2019)	United States	Cluster randomised control trial	Nurses and nursing assistants in nursing homes	30 Size: SMEs as a part of a larger organization All 30 long-term health care facilities belonged to one for-profit nursing home employer From each workplace between 24 and 89 direct care employees participated (M = 49.39, SD = 17.90)	931 at baseline (92.3% female, 7.7% male)	420 (Gender not reported)	511 (Gender not reported)
Leiter et al. (2011)	Canada	Cohort analytic (two group pre + post)	Registered nurses, registered psychiatric nurses, ward clerks, physicians and licensed practical nurses	41 units (8 intervention, 33 control) from 5 workplaces Size: Not reported (Assumed to be large: three district health authorities and two hospitals)	1173 at baseline (86% female, 11.8% male, 25 not responding)	262 (Gender not reported)	911 (Gender not reported)
Linzer et al. (2015)	United States	Cluster randomised control trial	Physicians, nurse practitioners, and physician assistants	34 Size: SME as a part of a larger organization After approval from site-specific Institutional Review Board (IRB) primary care clinicians at 34 primary care clinics were recruited	166 at baseline (51.8% female, 48.2% male)	83 (53.1% female, 46.9% male)	83 (50.6% female, 49.4% male)
Olson et al. (2016)	United States	Cluster randomised control trial	Home care workers in publicly funded programs	16 clusters Size: SME (micro) as part of a larger organisation 16 clusters (range 7–12 members) The clusters were created by the researchers to compare groups who received the intervention with groups who did not recruitment focused on HCWs represented by a union who cared for consumer-employer (CEs) enrolled in publicly funded programs managed by the Oregon Home Care Commission (OHCC). The OHCC encourages voluntary participation in further training with pay and other benefits Participants were employed by at least one public–private-pay CE	148 at baseline (89.9% female, 10.1% male)	74 (88% female, 12% male)	74 (90.5% female, 9.5% male)
Redhead et al. (2011)	United Kingdom	Randomised control trial	Mental Health Nurses (Qualified and Unqualified)	1 Size: SME as a part of a larger organization The study was conducted at a Low-secure unit (LSU) which is part of the range of mental health care provision available in England. A total of 79 nursing staff worked on the LSU from which participants were recruited	42 at baseline (60% female, 40% male)	22 (63.3% female, 36.4% male) Qualified staff: 12 (83.3% female, 16.7% male); Unqualified staff: 10 (40% female, 60% male)	20 (55% female, 45% male) Qualified staff: 9 (77.8% female, 22.2% male); Unqualified staff: 11 (36.4% female, 63.6% male)
Saffari et al. (2021)	Iran	Randomised control trial	Nursing Staff	4 Size: Not reported but assumed to be large (The intervention was conducted in four academic teaching hospitals in four mixed medical surgical intensive care units.)	207 at baseline (160 in all follow-ups, 72% female, 28% male)	155 (Gender not reported)	52 (Gender not reported)
Stansfeld et al. (2015)	United Kingdom	Cluster Randomised control trial	Employees and Managers of a mental health trust	1 Size: Large	350 employees at baseline (76% female, 24% male)	Employees: 283 (73.9 female, 26.1% male); Managers: 49	Employees: 67 (85% female, 15% male)
Tran et al. (2010)	Australia	Cohort analytic (two group pre + post)	Nurses	1 Size: Large	125 at baseline (88% female, 12% male)	74 (86.5% female, 13.5% male)	51 (94% female, 6% male)
Uchiyama et al. (2013)	Japan	Cluster Randomised control trial	Nurses	2 Size: Large	319 at baseline (98.7% female, 1.3% male)	183 (100% female)	218 (97.6% female, 2.4% male)
West et al. (2014)	United States	Randomised control trial	Physicians	1 Size: Large	74 at baseline (33.8% female, 66.2% male)	37 (32.4% female, 67.6% male)	37 (35.1% female, 64.9% male)
White and Winstanley (2010)	Australia	Randomised control trial	Nurses	17 Size: Not reported but could include SMEs as a part of a larger organization The RCT was situated in 17 green-field adult mental health facilities, in 9 participating locations, in public and private inpatient and community settings	186 (115 trainees + 71 nurses) at baseline (Gender not reported)	9 sites, 24 clinical supervision trainers (70.8% female, 29.2% male), 115 trainees (who received the intervention) and 43 unit staff	6 sites, 71 nurses (42% female, 58% male), and 11 unit staff
Van Woerkom (2021)	The Netherlands	Cluster randomised control trial	Nurses	1 Size: not reported (assumed to be large: 5 wards in one hospital)	95 (84.2% female, 15.8% male)	67 on 173 night shifts (Gender not reported)	28 on 70 night shifts (Gender not reported)

^*^Size of Organisation Definitions: Large: ≥ 250 employees; SME < 250 employees; Small: < 50 employees; Micro: < 10 employees; Varied (including SMEs); SME as a part of a larger organization

### Types of interventions

We built upon the classification developed by Fox et al. (2022) to categorize the different types of workplace interventions. To cover all types of interventions specific to the healthcare sector, we expanded the classification with two additional categories resulting in a total of six categories. We categorized the interventions based on our assessment of their most prominent intervention aspect, while being aware that many of these complex interventions also consisted of aspects of one or more of the other intervention group categories. Table 3 shows the details of the intervention characteristics of the 22 included studies by type of intervention.

**Table 3:** Intervention characteristics by intervention types

First author, year	Primary aim	Intervention details	Dose/duration of intervention	Follow-up—measurement points
Flexible work and scheduling changes				
Ali et al. (2011)	To assess the impact of weekend respite for intensivists on them and their patients	Daily coverage by a single intensivist in half-month rotations (continuous schedule) was compared with weekday coverage by a single intensivist, with weekend cross-coverage by colleagues (interrupted schedule). Weekend-covering intensivists could have other non-ICU clinical responsibilities during the weekdays, but not over the weekends they covered the ICU. In both schedules intensivists took call overnight from home	9 months Each site conducted the study for 9 months divided into three equal phases: thus, each ICU alternated twice between the two staffing schedules	Immediately after each half-month rotation
Garland et al. (2012)	To assess effects of around-the-clock intensivist presence on intensivists’ burnout	Compared two staffing models: the standard model where one intensivist staffed an ICU for 7 days, was present during daytime, and took calls from home at night and returned to ICU during night if necessary; and the shift work model, with one intensivist working 7-day shifts, while other intensivists covered the ICU at night	8 months Four blocks of eight weeks each. During each block one ICU with standard staffing and the other with intervention (shift work); each site alternated three times between staffing models	Immediately after each rotation
Job and task modifications				
Deneckere et al. (2013)	To evaluate the impact of the implementation of a quality improvement strategy (Care Pathways for 2 diseases: COPD and proximal femur fractures), compared with usual care, on interprofessional teamwork in an acute hospital	Development and implementation of Care Pathways involving restructuring of work processes and standardisation of procedures to avoid unnecessary variation with active goal setting within teams and clarification of roles. Three active components: (1) Formative evaluation of the teams ' performance before implementation with feedback report for each team (2) Evidence-based KIs for each team for the respective disease group couple with a workshop on the content of these KIs. (3) Training in pathway development for each study coordinator to develop the specific CP based on the findings of the formative evaluation	9 months	Immediately after implementation completion, i.e. 9 months post baseline
Gregory et al. (2018)	To measure the impact in primary care providers’ burnout of an implemented workload intervention that changed the work processes within primary care clinics	Change in work processes by introducing work teams consisting of two primary care providers and three Certified Medical Assistance, who jointly manage a panel of patients. This team was responsibility for the diagnosis, treatment, and care of a designated group of patients and was designed to reduce the workload on each team member. This model was compared to traditional work processes with a less team-oriented version of care applying dyads of staff	Permanent: Natural experiment study: changes were implemented permanently	3 months, 6 months after implementation
Linzer et al. (2015)	To assess if improvements in work conditions reduce clinician stress and burnout	Research staff facilitated discussions among clinicians and provided guidance on the type of intervention to select following a survey of working conditions. Selected in implemented interventions included a range of workplace changes, related to specific improvements in communication and discussion of issues especially among clinicians and staff; changes in workflow between staff; and targeted quality improvements programmes in response to clinician concerns	Permanent: Clinicians chose and implemented the changes that seemed most appropriate to them	Immediately after completion of intervention (12—18 months after baseline)
Redhead et al. (2011)	To evaluate the outcomes of a psychosocial intervention (PSI) training programme on the knowledge, attitudes, and levels of clinical burnout of qualified and unqualified nursing staff working in a low-secure mental health unit (LSU) and to assess evidence of implementation of PSI in practice	PSI training programme tailored for qualified staff (broad range of PSI including cognitive behavioural approaches for managing symptoms), and unqualified staff (focussed on understanding symptom related behaviours, relationship formation and help services users to cope with symptoms). Teaching sessions were supplemented by small group supervision, facilitated by the teaching team	8 months; qualified staff: 12 half-day sessions delivered over 8 months; unqualified staff: 8 half-day sessions	Immediately post last session
Saffari et al. (2021)	To compare the effects of a staged skill-based education programme (3 arms) focusing on improving professional knowledge and skills for enhancing ICU performance on ICU nurses’ stress and anxiety	15-page self-study booklet and a series of 90–120-min oral presentations by the researchers covering the scope of intensive care (e.g., definition, epidemiology and aetiology, risk factors, and clinical and economic outcomes) and management of the problem (i.e., 17 ventilator bundle components) Group 1 (control group): no intervention or education Group 2: only the self-study booklet without any participation in the oral presentation Group 3: oral presentations 14 days after completing the self-study booklet. Interactive, standardized, and mandatory oral presentations were held five times to ensure maximum attendance of the three nursing shifts Group 4: clinical teaching in the bedside after completing the self-study booklet and a series of interactive, standardized, and mandatory oral presentations	6 months Routine + Booklet: none; Routine + Booklet + Oral: interactive, standardised presentation held five times; Routine + Booklet + Oral + Supervision: interactive, standardised presentation held five times and clinical supervision, delivered over 6-month period (90–120 min)	Immediately after the intervention (6 months after baseline and 9 months after implementation (15 months after baseline) and 15 months after implementation (21 months after baseline)
White and Winstanley (2010)	To evaluate the impact of a skill-based clinical supervision (CS) training on the quality of nursing care, patient outcomes, and mental health and wellbeing of mental health nurses	Training of nurses as CS supervisors (practical CS sessions with direct feedback, each of which followed a linked program of theory-based seminars). During the year-long Intervention phase, each trainee supervisor conducted group supervision sessions at least once a month for 12 months with supervisees in their respective mental health service locations following explicit protocols (review progress and provide advice and support)	12 months Training course: 4 days, residential Supervision and monitoring by trainers: monthly over 1 year, approx. 12 group supervision sessions (45–90 min according to protocol)	Immediately after intervention (i.e. 12 months, plus or minus 2 weeks after baseline)
Relational and team dynamics interventions				
Jakobsen et al. (2017)	To evaluate the effect of a supervised group-based workplace physical exercise programme versus an alone unsupervised home-based physical exercise programme on psychosocial working environment factors among healthcare workers	Group-based and supervised strength training, during working hours at the hospital, using elastic bands, kettlebells and swiss balls in designated rooms located at or close to the respective departments. Supervised by experienced training instructors who ensured training progression. 5 group-based motivational coaching sessions with 5–12 participants in each session during working hours	10 weeks Intervention Group: Training: 5 × 10 min sessions per week for 10 weeks; 5 group motivational coaching sessions (30–45 min/per session) Control group: participants were instructed to exercise for 10 min, 5 days per week during leisure time using at least 4 exercises per session of the 10 different exercises shown in 3 posters	Immediately after intervention (i.e. 10 weeks past baseline)
Leiter et al. 2011, 2012	To investigate the effectiveness of a workplace civility intervention to improve social relationships (i.e., more civil and fewer uncivil interactions, and more respect). To study whether improved relationships be accompanied by improvements in employees’ burnout, turnover intentions, job commitment, satisfaction, trust in management, and absenteeism, such that these social relationships mediate the effect of the intervention on these broader outcomes	Stages of the intervention (CREW) 1: Preparation phase to introduce the concepts of civility and incivility to workers and management, and to introduce CREW as an inclusive process with explicit management endorsement 2: Baseline survey to identify levels of civility and organisational attitudes/behaviours for each work group. Facilitators are provided with a profile of their unit’s measured levels 3: Building of a learning community by organising a first meeting of facilitators and hospital leaders from different hospitals in the region for CREW training 4: Six months of facilitated weekly CREW meetings in groups of 10–15 employees of the same hospital unit 5: Three-month midpoint meeting of facilitators and hospital leaders for refresher and advanced CREW training with opportunities for sharing experiences in implementing CREW process 6: Final meeting of facilitators and hospital leaders at 6-month for sustainability training and community building. They receive results of their unit changes in relevant outcomes based on a follow-up survey	6 months Workers: Weekly meetings with structured exercises over 6 months Facilitators and hospital leaders: 3 meetings at baseline, 3 months and 6 months	Immediately after the intervention (i.e. 6 months after baseline and 6 months after the intervention, (i.e. 12 months after baseline)
Olson et al. (2016)	To determine the effectiveness of the COMmunity of Practice and Safety Support (COMPASS) Total Worker Health intervention for home care workers in relation to their perception of the quality of community of practice, health and safety behaviours and wellbeing	Researcher-led half-day workshop followed by 12 monthly peer-led meetings using scripted workbooks and supporting materials. Team leader manuals included additional instructions for peer leaders. Peer leaders received brief facilitator training at the beginning and midway through the program. The repeating monthly meeting routine involved a check-in (i.e., sharing current work and life status), educational lesson, goal setting, healthy meal break, WorkLife support (i.e., structured problem solving), and a reflection. Educational lessons alternated between safety and health or well-being topics. Teams could choose a schedule of readings, which they had to complete before each meeting. Each session included scripted group and individual goal setting. Intervention participants were provided individual and team “certification” incentives for meeting certain attendance and goal completion criteria	12 months Half-day workshop followed by 12 monthly peer-led meetings (Variable)	6 months after baseline (i.e. during the intervention), and immediately after the intervention (i.e. 12 months after baseline)
Participatory and enabling workplace change interventions				
Bourbonnais et al. (2011)	To assess the long-term effects of a workplace intervention designed to improve psychosocial work factors on mental health problems among health care professionals in an acute care hospital	Using a participatory approach, an intervention team, including healthcare professionals from three targeted care units and other important stakeholders from the hospital, developed suggestions for changes to reduce adverse psychosocial work factors and the best way to implement these changes. During a 4-month period, the intervention team met eight times for 3 h each	4 months	36 months (three years) after completion of intervention (Results from an earlier follow up 12 months after baseline—8 months after the intervention have been published in 2007, but these results are not presented in this review as the article did not match our search period that started 2010.)
Havermans et al. (2018)	To investigate the effectiveness of a digital platform to promote the use of work stress interventions at primary and secondary prevention levels and the level of implementation among healthcare workers	Online platform to direct organizations to psychosocial risk analysis, conducted in a participative manner and identifying organisational risk factors for work stress with guidance for determining an intervention while mastering implementation barriers. The platform contained a search engine with a broad selection of stress prevention interventions. The digital platform pages were structured in a stepwise approach: i) awareness raising; ii) assessment; iii) planning; iv) implementation; and v) evaluation	The intervention group was asked to implement the strategy within the first 6 months, but they had access to the strategy for 12 months. One member of each of the teams in the intervention group received a training in the use of the digital platform (Variable)	Immediately after the intervention (i.e. 6 months after baseline) and 6 months after implementation (i.e. 12 months after baseline)
Kossek et al. (2019)	To determine the effects of an intervention designed to increase job control and supervisor social support for work and nonwork issues on the stress levels of eldercare workers while taking into account their own family caring responsibilities	Participatory group training sessions to change work practices and processes to increase employees’ control over work time, supplemented by after-session work-improvement redesign activities. Supervisory training on supporting employees personal and family lives while also supporting good job performance, behavioural self-monitoring by leaders and coworkers The training was delivered by experienced trainers in organisational and leader development	4 months	2 months after the intervention (i.e. 6 months after baseline), 8 months after the intervention (i.e. 12 months after baseline) and 14 months after intervention (i.e. 18 months after baseline)
Stansfeld et al. (2015)	To investigate the feasibility of recruitment, adherence and effectiveness of an e-learning intervention for managers to improve employees’ well-being and reduce sickness absence	E-learning programme for managers included: 1) Pressure at work, the link with mental and physical ill health, relevance for addressing the issue; 2) How to proactively identify collective problems with their teams and find solutions; 3) Spot and approach employees with problems and find solutions; 4) Support individual employees with problems; 5) legal duty of care, avoidance of personal injury claims and HSE- compatible risk assessment; and 6) Critical reflection of their own management style in relation to increasing or reducing stress	3 months Delivered weekly to two weekly modules (six separate modules) with an introductory and follow-up face-to-face sessions	Immediately (i.e 3 months after baseline)
West et al. (2014)	To test the hypothesis that a facilitated physician small-group curriculum interventions results in improvement in well-being	The intervention involved 19 bi-weekly physician discussion groups incorporating elements of mindfulness, reflection, shared experience, and small-group learning during protected paid time. Groups were facilitated by specifically trained internal medicine physicians. The control group received 1 h of unstructured time every 2 weeks without clinical duties	9 months 19 sessions (1 h every 2 weeks)	3 follow-ups during the intervention (at 3, 6 and 9 months after baseline) and 2 follow-ups after the intervention (i.e. 3 and 12 months after the intervention)
Uchiyama et al. (2013)	To investigate the effects on mental health among nurses of a participatory intervention aimed at improving the psychosocial work environment in hospital settings	The participatory program for improving the psychosocial work environment was unit-based and focussed on active participation of employees with action planning. Subchief nurses served as key persons to facilitate activities within their units. The intervention included an intensive intervention period for the first 3 months and a consecutive implementation period for the following 3 months. The intensive intervention period consisted of 30-min group meetings with key persons to discuss intervention activities and barriers to implementation in respective unit. Key persons were also asked to complete task sheets after each meeting to specify the needs, progress and assist in workplace change activities 30-min individual interviews with each key person were conducted by the first author to provide advice on facilitating other staff activities in respective units. Then, each key person went back to their unit and shared necessary information with staff of their own units as follow-up tasks	6 months Key person meetings (30 min each) at least four times; Sessions 1–3 monthly + booster (30 min)	Immediately after the intervention (i.e. 6 months after baseline)
Changes in the physical work environment				
Cordoza et al. (2018)	To determine the effect on ICU nurse burnout of taking daily work breaks in a hospital-integrated garden compared to indoor-only breaks	Purposefully designed garden using natural features in close proximity to hospital units	6 weeks Daily (minimum 15 min)	Immediately after the intervention (i.e. 6 months after the intervention)
Emani et al. (2020)	To evaluate the impact of chromotherapy-based interventions on wellbeing and stress in ICU nurses”	Changing colour of decoration and work equipment in ICU, based on the principles of chromotherapy, coupled with three educational sessions on chromotherapy and individualized weekly consulting sessions on chromotherapy principles applying in personal life for three months. In addition, checklists of chromotherapy completed by participants with review by the researcher and weekly feedback	3 months 3 group sessions, then weekly individual sessions for three months	1 month after completion of intervention (i.e. 4 months after baseline)
Van Woerkom (2021)	To study the effectiveness of a napping facility and blue-light glasses on fatigue and wellbeing of nurses on night shift	Intervention group 1: Nurses (ICU) were given access to napping facility for 20 min Intervention group 2: Nurses (Paediatric care) were provided with light therapy glasses with integrated blue light LEDs. Nurses were instructed to wear the glasses for 30 min, between 2 a.m. and 4 a.m., right before they experienced a lack of energy Intervention group 3: Emergency care nurses with access to both interventions Control group: No intervention (Medium Care, Orthopaedics)	3 nights INT 1: 1 × 20 min nap per night over 3 night shifts. INT 2: Glasses worn for 30 min between 2 a.m. and 4 a.m (INT 1: 20 min. INT 2: 30 min)	Immediately after the intervention (i.e. Start and end of nightshift)
Improvement of employees’ mental health through changes in the way (patient) work is done				
Barcons et al. (2019)	1) to determine the effectiveness of an intensive multimodal training programme (MTP) with an Integrated Brief Systemic Therapy (IBST) approach for GPs designed to improve their management of mental health patients; and 2) to ascertain if the program could be also useful to improve GPs’ own burnout, job satisfaction and psychological well-being	Continuing-education course with group psychoeducational activities coordinated by the clinical psychologist guided by the psychotherapy model and the CCMs (team-based, multicomponent intervention)	9 weeks 9 weekly sessions (1 h per session, weekly)	10 months post-intervention
Tran et al. (2010)	To compare the nursing outcomes (job satisfaction, level of stress at work, job tension, and role ambiguity and role conflict) between nurses delivering a locally adapted team nursing model of care, known as shared care in nursing, with the existing patient allocation model of care	The team-nursing model of care consists of care delivered to a group of patients by a team of nurses and other staff with varying levels of education and skills, under the direction of a team leader. The model was locally adapted in consultation with clinicians, managers and administrators of the participating hospital. Attendance of clinicians in professional development sessions with educational courses, access to practice guidance and clinical supervised experience to enhance leadership skills, decision-making, delegation, empowerment and accountability, giving and receiving feedback, time management and mentoring of junior staff	Permanent	Immediately (i.e. 6 months after start of intervention)

#### Flexible work and scheduling changes

Interventions that focus on working time with regard to giving employees more possibilities to make changes in assigned work schedules or better possibilities to rest between shifts.

#### Job and task modifications

Interventions that implement enhanced work processes resulting from organisational, administrative and/or technical changes or increased professional competence.

#### Relational and team dynamic initiatives

Interventions that aim to increase the social relations at work through, e.g., team building activities.

#### Participatory and enabling workplace change interventions

Interventions that are developed with the participation of employees and their supervisors to tailor changes in the psychosocial work environment in response to a prior needs assessment. In contrast to the category ‘Job and task modifications’ the main aspect of this intervention type is on the participatory and enabling process for workplace changes without necessarily implementing these changes during the intervention period.

#### Changes in the physical work environment

Interventions that aim to improve employees’ mental health through changes in the physical work environment through e.g., better rest areas and calmer work environments.

#### Improvement of employees’ mental health through changes in the way (patient) work is done

Interventions that mainly focus on changes in the delivery of care for patients but at the same time aim to improve employees’ mental health outcomes through these care delivery changes.

### Number of follow-ups

In relation to follow-up, most studies (14 studies) reported one follow-up measurement, five studies had two follow-ups, two studies had three follow-up measurements, and one study had five follow-ups. Although most follow-up measures (13 studies) were taken immediately after completion of the intervention, nine studies reported longer-term results at one (Emani et al. 2020), six (Leiter et al. 2012; Gregory et al. 2018; Havermans et al. 2018), 10 (Barcons et al. 2019), 12 (West et al. 2014), 14 (Kossek et al. 2019), 15 (Saffari et al. 2021), and 36 months (Bourbonnais et al. 2011) post completion of the intervention. The duration of the interventions varied between three nightshifts (van Woerkom 2021), several weeks up to four months (Bourbonnais et al. 2011; Stansfeld et al. 2015; Jakobsen et al. 2017; Cordoza et al. 2018; Kossek et al. 2019; Barcons et al. 2019; Emani et al. 2020), six months (Leiter et al. 2012; Uchiyama et al. 2013; Havermans et al. 2018; Saffari et al. 2021), eight to nine months (Redhead et al. 2011; Ali et al. 2011; Garland et al. 2012; Deneckere et al. 2013; West et al. 2014), 12 months (White and Winstanley 2010; Olson et al. 2016), and permanent changes to working structures and procedures (Tran et al. 2010; Linzer et al. 2015; Gregory et al. 2018).

### Quality appraisal results

Table 4 shows a summary of the quality appraisal of the studies. Further details can be found in the appendix 2. Overall, more than three-quarters of the 22 studies (16 studies) were assessed to have strong or moderate methodological quality. In relation to the appraisal of the six QATQS quality domains, none of the studies were rated as weak regarding the data collection method and only one study (Cordoza et al. 2018), was considered weak regarding the study design, which reflects the strict inclusion criteria allowing for controlled studies only and for those with validated outcome measurement.

**Table 4:** Summary of quality assessment of the selected studies

Quality of the selected studies		
Weak	Moderate	Strong
Cordoza et al. (2018)	Ali et al. (2011)	Garland et al. (2012)
Gregory et al. (2018)	Barcons et al. (2019)	Jakobsen et al. (2017)
Kossek et al. (2019)	Bourbonnais et al. (2011)	Linzer et al. (2015)
Leiter et al. (2011)	Deneckere et al. (2013)	Saffari et al. (2021)
Tran et al. (2010)	Emani et al. (2020)	Stansfeld et al. (2015)
White and Winstanley (2010)	Havermans et al. (2018)	Uchiyama et al. (2013)
	Olson et al. (2016)	West et al. (2014)
	Redhead et al. (2011)	
	Van Woerkom (2021)	

Most limitations were found regarding the lack of blinding the outcome assessors to the intervention status and the participants to the research questions (nine studies were rated as weak in this domain), and regarding high rates of undocumented withdrawal and dropout (seven studies). Lack of blinding may result in an overestimation of the intervention effect, whereas differential dropout of participants, commonly the less healthy individuals, is likely to result in an underestimation of the intervention effect.

### Outcome results

Table 5 shows a summary of the effectiveness results for the primary outcomes of this review (mental wellbeing, stress, burnout, depression and anxiety symptoms) and for the secondary outcomes (psychosocial working conditions and absenteeism) for the six intervention types. The table also shows the level of evidence for each of the six intervention types. The category ‘mental wellbeing’ included constructs classified by the authors such as ‘vitality' and 'mental health’; the category ‘stress’ included constructs such as ‘unspecific distress’, ‘secondary stress’, ‘perceived stress’, ‘psychological distress’, ‘work-related tension’ and ‘work-related threat’. ‘Burnout’ encompassed ‘emotional exhaustion, ‘depersonalisation’, ‘cynicism’, ‘personal accomplishment’ and ‘work-related, client-related and personal burnout’. Depression symptoms included general depressive symptoms. Anxiety symptoms comprised general anxiety symptoms and state and trait anxiety. Detailed results can be found in appendix 3 and 4.

**Table 5:** Overview of outcome measures and level of effectiveness in the six types of organisational workplace interventions

Studies (quality)	Wellbeing	Stress/distress	Burnout	Depression	Anxiety	Secondary outcome work characteristics/absenteeism	Statistically sig. impact (50% of primary outcomes were sig. improved)	Consistency more than 75% of studies within an intervention category had a statistical sig. impact	Level of evidence: ‘strong’ (consistent findings in multiple high-quality studies), ‘moderate’ (consistent findings in one high-quality and/or multiple moderate-quality studies), ‘weak” (consistent findings in one moderate-quality and/or multiple low-quality studies), ‘insufficient’ (only one study available or inconsistent findings or null findings in multiple studies)
Flexible work and scheduling changes (2 studies)									
Ali et al. (2011) (moderate)		Improve US-scale	Improve US-scale			Improve (work-life balance)	Yes	100% (2 of 2)	Moderate
Garland et al. (2012)			Improve MBI (EE *)			Improve (work-life balance; job overload) Increase in role uncertainty	Yes		
Job and task modifications (6 studies)									
Deneckere et al. (2013) (moderate)			Improve UBI				Yes	83% 5 of 6	Strong
Gregory et al. (2018) (weak)			Improve MBI (only for EE *)			Improve workload (only 1 follow-up)	Yes		
Linzer et al. (2015) (strong)		No effect 5-point scale (Motowidlo et al. 1986)	Improve (in the individual focused analysis) 5-items-scale				Yes		
Redhead et al. (2011) (moderate)			Improve MBI (only for depersonalization scale and only for qualified nurses)				Yes		
Saffari et al. (2021) (strong)		Improve PSS-14			Improve STAQ** (improved state anxiety, but not trait anxiety)		Yes		
White and Winstanley (2010) (weak)	No effect SF8		No effect MBI	No effect GHQ 28	No effect GHQ 28		No		
Relational and team dynamics interventions (3 studies; 4 articles)									
Jakobsen et al. (2017) (strong)	Improve SF-36 (vitality)			No effect SF-36 (mental health)		Increase in work pace No effects for emotional demands, influence at work, sense of community at work, social support from supervisor	*Yes*	66% (2 of 3)	Insufficient
Leiter et al. (2011) (weak)			Improve MBI (only in cynicism (depersonalization) subscale, not in emotional exhaustion subscale)			Improve (self-reported absence)	Yes		
Olson et al. (2016) (moderate)	No effect SF-12						No		
Participatory and enabling workplace change interventions (6 studies)									
Bourbonnais et al. (2011) (moderate)		No effect Abridged version of PSI	Improve CBI			Improve (psychological demands, effort-reward imbalance, quality of work, emotional demands)	Yes	50% (3 of 6)	Insufficient
Havermans et al. (2018) (moderate)		Improve DASS-21				No effects (psychological demands, social support, autonomy)	Yes		
Kossek et al. (2019) (weak)		Improve PSS 4 items and Psychological Distress, 6 items					Yes		
Stansfeld et al. (2015) (strong)	No effect WEMWBS	No effect GHQ-12				No effects (absenteeism, supervisor relationship, supervisor support)	No		
West et al. (2014) (strong)		No effect PSS	Improve MBI	No effect Validated 2-question approach			No		
Uchiyama et al. (2013) (strong)				No effect CES-D		Improve (coworker support, goals) No effects for effort and reward, for demands and supervisor support, borderline sig for job control No effects for the other aspects in the QWC: efficiency, participatory management, competence development, work climate, leadership, feedback	No		
Changes in the physical work environment (3 studies)									
Cordoza et al. (2018) (weak)			Improve MBI				Yes	100% (3 of 3)	Moderate
Emani et al. (2020) (moderate)		Improve secondary post-traumatic stress 10 items in ProQol***	Improve Burnout 10 items in ProQoL***				Yes		
Van Woerkom (2021) (moderate)	Improve EFQ						Yes		
Improvement of employees’ mental health through changes in the way (patient) work is done (2 studies)									
Barcons et al. (2019)	No effect BPRS		No effect MBI				No	0% (0 of 2)	Insufficient
Tran et al. (2010)		No effect SIG with two subscales: pressure at work and work-related threat				No effect (role conflict and ambiguity)	No		
Number of measurements (improve/no effect) *Negative effects*									
	6 (2/4) *0*	10 (5/5) *0*	13 (11/2) *1*	4 (0/4) *0*	2 (1/1) *0*	10 (6/4) *2*			
Success rate									
	33%	50%	85%	0%	50%	60%			

Scales: Wellbeing—SF-8 short form health-related quality of life, SF-36 36-item short form health survey, SF-12 12-Item short form health survey, *WEMWBS* Warwick Edinburgh Mental Wellbeing Scale, *EFQ* everyday feelings questionnaire, *BPRS* brief psychiatric rating scale, Stress/Distress—US-Scale derived from the National Study of the changing workforce, *PSS-14* perceived stress scale 14-item, *DASS-21* depression anxiety stress scales-21 items, *PSS-4* perceived stress scale 4-item, psychological distress 6-item, *GHQ-12* general health questionnaire 12 items, *PSS* perceived stress scale, *ProQol* professional quality of life, *SIG* stress in general, Burnout—MBI Maslach burnout inventory, *EE* emotional exhaustion (one of the three subscales of the Maslach burnout Inventory), *UBI* Utrecht burnout inventory, *CBI* Copenhagen burnout inventory, Depression—GHQ-28 28-item general health questionnaire, *CES-D* center for epidemiologic studies depression scale, Anxiety—*STAQ* state-and trait anxiety questionnaire, secondary outcome—*QWC* quality work competence questionnaire

#### Flexible work and scheduling changes (2 studies)

This category consisted of two articles, one assessed to have moderate quality (Ali et al. 2011) and one to have strong quality (Garland et al. 2012). Both articles investigated if changes in work schedules, reducing continuous work and giving physicians more time to rest, had effects on mental health and other outcomes. In relation to primary outcomes, both studies reported improvements in burnout. In addition, one study (Ali et al. 2011) reported less distress. However, due to the fact that only one of the studies was of high quality, we assessed the level of evidence for the effectiveness as moderate. Regarding the secondary outcomes, both studies found improvements in work-life balance and one study (Garland et al. 2012) evidenced a decrease in the overall job overload, however, also increases in role uncertainty.

#### Job and task modifications (6 studies)

The studies in this category consisted of two studies of strong quality (Linzer et al. 2015; Saffari et al. 2021), two of moderate quality (Redhead et al. 2011; Deneckere et al. 2013) and two of weak quality (White and Winstanley 2010; Gregory et al. 2018). The studies investigated the effects of job and task modification interventions to enhance work processes or trainings to increase professional competence. There was high consistency of significant findings on mental health outcomes among these six studies and effects were found in more than one study with high quality. We therefore assessed the level of evidence for mental health outcomes for this category as strong.

Almost all studies in this category reported positive changes in at least one of the measured primary outcomes; the findings were particularly consistent for burnout. Of the five studies that measured burnout (White and Winstanley 2010; Redhead et al. 2011; Deneckere et al. 2013; Linzer et al. 2015; Gregory et al. 2018) four reported improvements, although Redhead et al (2011) only found improvements of the training intervention in qualified nurses, while the training program led to non-significant increased burnout in unqualified nurses. One study (White and Winstanley 2010) focussing on training psychiatric nurse supervisors in clinical supervision found no improvement in burnout neither for the trained supervisors nor their supervisees. Of the two studies (Linzer et al. 2015; Saffari et al. 2021) that measured stress, one study evidenced no effect of the intervention (Linzer et al. 2015), whereas the other one (Saffari et al. 2021) identified an improvement of stress after 6 and 15 months, which did not sustain after 21 months. Two studies (White and Winstanley 2010; Saffari et al. 2021) measured anxiety, one study (White and Winstanley 2010) found no effect, while one study (Saffari et al. 2021) found effects in the first two follow-up measurements but not in the final follow-up measurement after 21 months. Only one study (White and Winstanley 2010) measured wellbeing and symptoms of depression but found no impact of the intervention on these outcomes. Secondary outcomes were only investigated by one study. This study (Gregory et al. 2018) focussing on a workload intervention that changed the work process within primary care clinics found improvements in workload, however this improvement was only significant after 3 months and no longer after 6 months.

#### Relational and team dynamic initiatives: (4 articles, 3 studies)

Three studies, with one study published in two articles (Leiter et al. 2011, 2012; Olson et al. 2016; Jakobsen et al. 2017), targeted work groups and the ways workers interact and work together. One study was of strong quality (Jakobsen et al. 2017), one of moderate quality (Olson et al. 2016) and one of weak quality (Leiter et al. 2011, 2012). The consistency of findings showing a statistically significant impact across these 3 studies was below 75% and we therefore assessed that the level of evidence for mental health outcomes for this category as insufficient (Leiter et al. 2011, 2012; Olson et al. 2016; Jakobsen et al. 2017).

One study aimed at enhancing the community of practice and wellbeing of traditionally isolated home care workers by bringing them together in groups for health and safety education and peer support with team building activities did not demonstrate improvements in mental health and wellbeing (Olson et al. 2016). The study by Jakobsen et al. (2017) conducted with female hospital workers compared guided physical exercise with peers at work supplemented by coaching sessions during work hours with a control group of workers exercising alone at home during leisure time. They found an improvement in wellbeing in the intervention group that exercised at work in comparison to the control group exercising at home (Jakobsen et al. 2017). With regard to secondary outcomes, they found no improvements, but an increase in perceived work pace for the exercise–at-work-group, which may be explained by an increased demand to work faster to compensate for the time dedicated to exercise (Jakobsen et al. 2017).

The study by Leiter et al. (2011, 2012) introduced incivility training to improve the quality of working relationships, to increase civility and to decrease incidents of incivility. After the completion of the intervention after 6 months, the intervention was effective in reducing burnout (cynicism but not emotional exhaustion). Self-reported absenteeism was also significantly reduced by over one third from 0.88 days per month to 0.46 days per month in the intervention group, whereas absences remained fairly stable in the control group (0.86 to 0.83). However, the effect for absenteeism was not sustained at 12 months follow-up (Leiter et al. 2012).

#### Participatory and enabling workplace change interventions: (6 studies)

Six studies investigated participatory interventions that aimed at making changes in the psychosocial work environment based on the needs identified by employees and their supervisors (Bourbonnais et al. 2011; Uchiyama et al. 2013; West et al. 2014; Stansfeld et al. 2015; Havermans et al. 2018; Kossek et al. 2019). Three studies were assessed as strong quality (Uchiyama et al. 2013; West et al. 2014; Stansfeld et al. 2015), two as moderate quality (Bourbonnais et al. 2011; Havermans et al. 2018) and one as weak quality (Kossek et al. 2019). Despite some positive outcomes in some of the studies, the consistency of studies with statistical impact across the 6 studies was below 75%. We therefore assessed that the level of evidence for mental health outcomes for this category as insufficient. Overall, the findings were mixed for both the primary and the secondary outcomes. The two studies that measured burnout (Bourbonnais et al. 2011; West et al. 2014) evidenced improvements following the intervention. Both interventions involved employees in the development of suggestions for changes in the psychosocial working environment, one intervention (West et al. 2014) included facilitated physician small-group discussion meetings, the other intervention (Bourbonnais et al. 2011) consisted of a participatory approach in hospital units. Of the five studies that measured stress (Bourbonnais et al. 2011; West et al. 2014; Stansfeld et al. 2015; Havermans et al. 2018; Kossek et al. 2019), only one study (Havermans et al. 2018), that investigated the effectiveness of a digital platform-based implementation strategy, found an overall effect on stress which was mainly explained by an increase in stress in the control group rather than a reduction of stress in the intervention group. Another study (Kossek et al. 2019) found a positive effect for stress for caregivers in eldercare nursing facilities who had additional care responsibilities in their family in addition to their care work. However, these effects were not found at the first follow up after 6 months, but at the second and third follow up after 12 and 18 months. Of the two studies that measured wellbeing (Uchiyama et al. 2013; Stansfeld et al. 2015), none found an effect. Also, the two studies that measured symptoms of depression (Uchiyama et al. 2013; West et al. 2014) found no effect. Four studies measured, whether the intervention resulted in an improvement of psychosocial working conditions (secondary outcomes) (Bourbonnais et al. 2011; Uchiyama et al. 2013; Stansfeld et al. 2015; Havermans et al. 2018), of which two studies (Bourbonnais et al. 2011; Uchiyama et al. 2013) found positive changes.

Uchiyama et al (2013) measured several aspects of the psychosocial working environment, but only found an effect for co-worker support. Bourbonnais et al (2011) found effects for psychological demands, effort-reward imbalance, quality of work, physical demands and emotional demands. The remaining two studies found no effects in psychosocial work environment aspects, such as job demands, autonomy and supervisor relationship and supervisor support (Stansfeld et al. 2015; Havermans et al. 2018). Stansfeld et al. (2015) also did not find reductions in absenteeism.

#### Changes in the physical work environment (3 studies)

Three studies focussed on environmental workplace changes (Cordoza et al. 2018; Emani et al. 2020; van Woerkom 2021). Studies in this intervention category were rated either moderate methodological quality (Emani et al. 2020; van Woerkom 2021) or weak methodological quality (Cordoza et al. 2018). There was 100% consistency among these three studies with regard to their significant impact on mental health outcomes (Cordoza et al. 2018; Emani et al. 2020; van Woerkom 2021). However, due to the quality of the studies we assessed the level of evidence for this category as moderate. Each of the three studies showed positive effects of the intervention on mental health outcomes either on burnout (Cordoza et al. 2018; Emani et al. 2020), on wellbeing (van Woerkom 2021) or on secondary traumatic stress (indirect trauma that can occur when exposed to traumatised or terminally ill patients) (Emani et al. 2020). Physical work environment interventions included facilitation of daily breaks in a garden area compared to breaks in an indoor break room for nurses (Cordoza et al. 2018), access to both a napping facility and blue light therapy glasses for nurses during night shifts (van Woerkom 2021), changes in the colour of decoration in intensive care units (ICU) following the principles of chromotherapy coupled with educational sessions for the application of these principles in personal life (Emani et al. 2020).

#### Changes in the way patient work is done (2 studies)

Two studies, one of moderate (Barcons et al. 2019) and one of weak methodological quality (Tran et al. 2010) investigated changes in models of care and work processes, designed to improve the delivery of care to patients, on the mental health and wellbeing of the care providers. None of the studies showed significant changes in mental health indicators of the care providers (Tran et al. 2010; Barcons et al. 2019). Due to the lack of findings for mental health outcomes in both studies, we assessed the level of evidence for this category as insufficient. One study (Barcons et al. 2019) found no significant effect of an intensive multimodal training programme based on a collaborative care model on burnout, and psychological wellbeing in GPs, although the study group showed enhancements in patient care performance indicators. Tran et al. (2010) compared two models of providing nursing care, the Team Nursing Model with teams of healthcare professionals with varying levels and skills delivering care to a number of patients overseen by one registered nurse and the traditional Patient Allocation model with one registered nurse being responsible for the total care of a number of patients. No intervention effects of the Team Nursing Model were evident for the primary outcomes perceived stress and tension levels, nor for the secondary outcomes role conflict or job ambiguity (Tran et al. 2010).

## Discussion

This systematic review set out to assess the effectiveness of organisational interventions to enhance healthcare workers’ mental health and wellbeing with a specific focus on SMEs and intervention types. We identified 22 controlled studies based on 23 articles, most of them of strong to moderate methodological quality, which gives satisfactory confidence in the results. The categorisation of organisational interventions allowed the careful assessment of the effectiveness of specific types within the wide range of organisational interventions. In general, 15 of the 22 included studies (68%) showed improvements in at least one of the primary outcomes mental wellbeing, stress, burnout, symptoms of depression or symptoms of anxiety, respectively. We found considerable variation in the consistency of results by intervention type. The number of studies addressing the secondary outcomes, psychosocial working conditions and absenteeism, was low precluding any definite conclusions.

### Level of evidence by type of intervention

We found a strong level of evidence for the intervention category *Job and task modifications* and a moderate level of evidence for the categories *Flexible work and scheduling* and *Changes in the physical work environment.* For the remaining three categories of intervention types the existing evidence was insufficient for an assessment of the level of evidence, either because of mixed results (*Participatory and enabling workplace change interventions* and *Relational and team dynamic initiatives*) or because none of the studies in this category showed effects (*Improvement of employees’ mental health through changes in the way (patient) work is done*).

The six studies in the category *Job and task modifications showed* a clear pattern of positive effects in almost all of the studies. Four studies found positive outcomes for burnout, while one study found positive effects for stress and anxiety. However, it is noteworthy to flag, that one study found positive effects on burnout of their competency training programme to handle patients with severe mental illness for qualified nurses only but not for unqualified nurses (Redhead et al. 2011). This points to the fact, that professional training needs to be tailored to the particular competencies of the participants.

Both studies in the category *Flexible work and scheduling* found improvements in burnout and one study also found improvement in stress. Although we only identified two studies for this category, the included studies fulfilled our criteria for moderate level of evidence, since one of the studies was of strong quality. The positive effects of interventions giving employees more flexibility in their work schedules or more time to rest between shifts has also been found in a recent overview review of organisational workplace interventions (Aust et al. 2023).

The three studies in the category *Changes in the physical work environment* found improvements in mental health outcomes including wellbeing, stress and burnout. The studies supported employees in achieving better restitution during rest breaks through providing a garden, napping facilities or a chromotherapy-based intervention. In all of these studies, these specific work environment changes were provided by the workplace (i.e. the garden was established, the napping facilities were provided, and the colour panels were installed). Although the use of chromotherapy principles is controversial and needs further research, all studies provided real changes in the physical work environment and the results show that employees experienced improvements in mental health outcomes.

### Effectiveness by primary outcome

In relation to primary outcomes, most studies measured burnout, which also was the outcome that had the highest percentage of positive effects (11 of 13 studies, 85%). Stress was measured by 10 studies of which five studies found an effect (50% positive effects) and wellbeing was measured by seven studies of which two studies found a positive effect (29% positive outcomes). Only five studies measured symptoms of depression and anxiety. None of the four studies that measured depressive symptoms found an effect. Among the two studies that measured anxiety, one found a positive effect, although only for state anxiety and not for trait anxiety. Our findings are similar to a recent realist review that investigated workplace-based organisational interventions to promote mental health and happiness among health care workers, which found that the most common construct measuring mental health was burnout followed by stress (Gray et al. 2019).

The differences in effects for the different mental health outcomes might be due to a number of reasons including their prevalence and severity in a working population, but also in the way they were measured. Among the five primary outcomes we were interested in in this review, most consistency in the used measurement tools was found for measuring burnout. Eight of the 13 studies that measured burnout used the Maslach Burnout Inventory (MBI), which is a well-validated tool in workplaces including healthcare. However, for other mental health outcomes, the use of measurement instruments and operationalisations within one construct varied considerably. For example, among the 10 studies that measured stress, only three studies used a similar measurement tool (a version of the Perceived Stress Scale (PSS)), and among the six studies that measured wellbeing each study measured it differently. Some scales that were used to measure mental wellbeing do not measure the positive components of mental health and one study used the Brief Psychiatric Rating Scale (BPRS) to measure mental wellbeing which is designed to assess psychopathology in several psychiatric disorders, meaning that what was measured under the term “wellbeing” were in fact very different phenomena. Some measurement tools might not be appropriate to pick up changes among mostly healthy (working) participants. This might also be the reason why no changes were found in the four studies that measured symptoms of depression. For example, Uchiyama et al. (2013) who did not find an effect for symptoms of depression using the Center for Epidemiologic Studies Depression Scale (CES-D) discussed that this scale might not have been able to measure the milder reactions that participants might have experienced like irritation, anger or anxiety.

The way a certain outcome is measured might also have implications for the consistency of findings within one study. In the 11th Revision of the International Classification of Diseases (ICD-11) burnout is defined as “a syndrome conceptualized as resulting from chronic workplace stress that has not been successfully managed” (World Health Organization 2022). It would therefore be plausible to expect that interventions that are effective in reducing burnout also are effective in reducing stress. However, that is not always the case. In three studies (Bourbonnais et al. 2011; West et al. 2014; Linzer et al. 2015), the authors found an improvement in burnout but not in stress. One reason for this lack of connection might be that the scales used for the measurement of stress and burnout differ in their focus on work. Most of the stress scales used in the studies identified in this review measure experiences of exhaustion without a specific reference to work. They therefore do not measure “chronic workplace stress”. Contrary, burnout scales typically ask specifically about experiences due to work. As the aim of organisational level workplace interventions is to change conditions at work, burnout scales might be more sensitive to detect if these workplace changes affect employees’ mental health experience. However, it may also play a role that burnout is typically measured with three sub-scales (in the MBI, but also in the Copenhagen Burnout Inventory) which increases the chances for finding effects. Although the disconnection between stress and burnout outcomes in this review is only based on three studies, the already mentioned overview review of organisational level intervention points in the same direction. The overview review found strong quality of evidence for interventions aiming to reduce burnout while the evidence for reviews that investigated the effects of organisational level interventions on stress was inconclusive due to contradictory results (Aust et al. 2023).

### Effectiveness by secondary outcomes (psychosocial work environment and absenteeism)

Changes in the psychosocial work environment was included as a secondary outcome to reflect their role as intermediary outcomes of organisational interventions, which, by their nature, are targeted at modifying the psychosocial work environment (Aust et al. 2023). Assessing changes in psychosocial working conditions may also be a relevant measure in terms of successful training transfer following a supervisor training (Nielsen and Shepherd 2022) or an actual implementation of workplace changes following a participative intervention to improve the work environment. Of the 10 studies that investigated the impact of the intervention on psychosocial work environment variables, six found positive changes, especially in the dimensions work-life balance and work demands (Leiter et al. 2011, 2012; Bourbonnais et al. 2011; Ali et al. 2011; Garland et al. 2012; Uchiyama et al. 2013; Gregory et al. 2018). Only two studies investigated the secondary outcome absenteeism (Leiter et al. 2011, 2012; Stansfeld et al. 2015). One study (Stansfeld et al. 2015) found no effect, while the other (Leiter et al. 2011) did immediately after the intervention, however at the 6 months follow up it had returned to the pre-intervention level (Leiter et al. 2012). Five of the positive outcomes in psychosocial work environment aspects were accompanied by positive outcomes in one or more of the primary outcomes (especially burnout) (Leiter et al. 2011; Bourbonnais et al. 2011; Ali et al. 2011; Garland et al. 2012; Gregory et al. 2018), which could indicate that changes in the working environment contribute to changes in mental health (Stansfeld and Candy 2006). Almost all of these studies belong to the categories “Flexible work and scheduling changes”, “Job and task modifications” and “Participatory and enabling workplace change interventions” which are all categories that include workplace changes or try to initiate them through participatory processes. However, two studies (Jakobsen et al. 2017; Havermans et al. 2018) found positive effects in the primary outcomes without accompanying positive outcomes in psychosocial work environment aspects, showing that organisational interventions can have direct beneficial effects on mental health measures and workplace changes are not always mediating this relationship. In two studies (Garland et al. 2012; Jakobsen et al. 2017) the intervention led to negative effects in psychosocial work environment aspects, but these unintended effects seem avoidable by supporting employees better during the change process.

### Interventions introducing actual changes might be more successful

Our results suggest that intervention types that consist of actual changes, i.e., where the change in the work environment constitutes the intervention, have a higher chance of producing positive mental health and wellbeing effects than other types of organisational interventions. The intervention types “Job and task modifications”, “Flexible work and scheduling changes”, and “Changes in the physical work environment” all test the effects of implemented changes at the workplace and show a pattern of rather positive outcomes. This finding is in line with a systematic review by DeChant et al. (2019) on the effectiveness of organisational interventions on physician burnout that found that the largest benefits for burnout were found for interventions focussing on team-based care and improved work processes. It also corresponds with a study among more than 20 000 nurses and physicians in the United States who ranked interventions that provided real workplace changes such as sufficient staffing, control over workload and the possibility to take breaks without interruptions as most important for improving their wellbeing (Aiken et al. 2023).

In comparison, the study outcomes in the category “Participatory and enabling workplace change interventions” were more mixed. Participatory and enabling interventions commonly increase the competence of supervisors and workers to initiate, develop and implement work changes and have therefore the potential to lead to positive effects in increased control and improved working conditions, which both can contribute to better mental health outcomes (Stansfeld and Candy 2006). However, as mentioned above, these interventions also require that employees and their supervisors are interested in and have the competencies for making these changes as well as have the resources to actively participate in these activities. Participatory and enabling interventions might therefore more often be confronted with implementation barriers than other intervention types (e.g. lack of leadership support, limited resources). In fact, the three participatory and enabling interventions included in this review that were less successful with finding positive effects, report difficulties in implementing the interventions (Uchiyama et al. 2013; Stansfeld et al. 2015; Havermans et al. 2018). Failed or limited implementation might result in less positive outcomes as seen in this review (Uchiyama et al. 2013; Stansfeld et al. 2015; Havermans et al. 2018) and as seen in another study conducted in a hospital can even lead to negative effects (Aust et al. 2010). Our findings are in line with other reviews that assessed the effects of participatory workplace interventions that aim to increase employees’ control: While many studies have shown that these types of intervention can lead to positive effects for employees’ health, the evidence is not entirely consistent which is often due to incomplete implementation (Aust et al. 2023). Nevertheless, these interventions can lead to sustained effects as was shown in the study by Bourbonnais et al (2011) who found effects even three years after the intervention.

### Sustainability

From a population health perspective, organisational interventions have been discussed as a strategy with potential for sustainable impact on mental health, as they incorporate favourable working conditions into the organisational structure and procedures with possible effects for current but also for future workers even if individual workers leave the job (Greiner 2012; LaMontagne et al. 2019). Although most studies measured the immediate intervention effect, this review included studies that are providing outcome measures at medium-term (3–12 months after completion) and long-term (longer than 12 months after completion). The longest follow-up time (3 years) was reported by Bourbonnais et al (2011). This participatory intervention for workplace changes led to sustained decreases in burnout and several psychosocial working conditions after 12 months, as reported in an earlier publication (Bourbonnais et al. 2006). More importantly, these changes were sustained after three years (Bourbonnais et al. 2011). Also, West et al (2014) reported sustained improvements (12 months) in burnout following a participatory workplace change intervention with physicians. However, other findings on long-term effects are mixed. For example, Saffari et al (2021) reported that although the stress and anxiety scores decreased after 6 months and 15 months following a skill-based educational programme for ICU nurses, there was no significant reduction in the third follow-up at 21 months. The authors recommend continuous training to maintain the effectiveness over time. The study by Leiter et al. 2012 found that long-term effects of their civility intervention were different for different outcomes including continued improvements for civility, incivility, workplace distress and job attitudes, while the improvements in absenteeism were not maintained at the second follow-up 6 months after the intervention.

### Work groups in healthcare

The review included a range of occupations in a variety of clinical and community settings at different levels, however, the majority of participants were nurses and physicians within emergency and intensive care or mental health units. Most studies were conducted in hospitals, perhaps due to the ease of access to participants. Similarly, a recent overview review found that more than half of the 52 reviews of organisational interventions to improve the psychosocial work environment were conducted in large organizations in the healthcare sector (Aust et al. 2023). This is in line with recent reviews about workplace interventions among health care workers, that found a predominance of studies conducted among nurses and physicians in hospital settings (Gray et al. 2019; Fadel et al. 2023). Nevertheless, several occupational groups in healthcare were not represented in our review, such as first responders, dentists, orthodontists, midwives, physiotherapists, many of them potentially working in smaller organisations, pointing again at the lack of research in this area.

### SMEs

We found no conclusive results in relation to our review question about the effectiveness of particular types of workplace mental health interventions in SMEs. No study specifically focussed on SMEs, although some studies included SMEs in their sample without specifically analysing their data by size of organisation (Redhead et al. 2011; Linzer et al. 2015; Olson et al. 2016; Kossek et al. 2019; Barcons et al. 2019). In all of the aforementioned studies, the SMEs were a part of or attached to a larger overarching organisation that most likely provided access to mental health support, training or resources for work environment improvements. Therefore, it still needs to be shown that interventions to improve employee's mental health outcomes through organisational interventions are also effective in independent SMEs without access to this type of additional support. Only three of the studies that included SMEs (Redhead et al. 2011; Linzer et al. 2015; Kossek et al. 2019) found positive effects (on burnout or stress), while almost all of the 11 studies conducted in large organisations found positive effects (Tran et al. 2010; Bourbonnais et al. 2011; Garland et al. 2012; Deneckere et al. 2013; Uchiyama et al. 2013; West et al. 2014; Stansfeld et al. 2015; Jakobsen et al. 2017; Cordoza et al. 2018; Havermans et al. 2018; Emani et al. 2020). Conducting organisational interventions in SMEs might therefore be even more challenging than in larger organisations.

### E-interventions versus face-to-face interventions

Two of the 22 included studies applied an intervention that was delivered solely via an internet platform, including training in psychosocial risk assessment for supervisors and participatory development of workplace changes (Stansfeld et al. 2015; Havermans et al. 2018). Only one of them (Havermans et al. 2018) found a reduction of stress levels, and both studies did not find improvements in psychosocial working conditions following the intervention as hypothesised. E-mental health interventions have been discussed as the future of occupational mental health interventions, due to their easy accessibility, the flexibility of timing for the participant and the cost-effective delivery at a large scale (Lehr et al. 2016). Although a recent systematic review and meta-analysis of occupational e-mental health interventions in the workplace identified significant mental health improvements in employees (Phillips et al. 2019), most of these were targeted at the individual, while the effectiveness of organisational mental health and wellbeing e-interventions still needs to be shown. In general, it may be difficult to initiate a participatory workplace change process through an e-intervention or for management to introduce modified work processes, role responsibilities, cooperation or models of care delivery. While Stansfeld et al (2015) suggested, in reflection of their study results, to place more emphasis on experiential and active learning and affective engagement for managers to bring about workplace changes, more research is needed to identify which elements of organisational interventions can successfully be implemented through e-interventions and which elements require face-to-face interventions.

### Strengths and limitations

The present review only considered studies published by July 9, 2021. We did not update the search further, because we did not want to include studies that were conducted under the extraordinary circumstances due to COVID-19. The three identified studies that were published in 2020 and 2021 (Emani et al. 2020; Saffari et al. 2021; van Woerkom 2021) reported about interventions conducted before the pandemic. Despite this limitation, we believe that our review contributes with a valuable overview of experiences with organisational interventions in the healthcare sector.

A very broad approach was used to identify organisational interventions in the healthcare sector resulting in a variety of intervention types conducted across different occupational groups and mixed teams. Although this heterogeneity did not allow firm conclusions about specific interventions for specific groups, it provided the opportunity to show the large variety of organisational intervention approaches. Dividing the interventions into six different categories helped to better identify types of interventions that seem to be more likely to reach positive effects than others. Another strength is, that we looked at a variety of mental health outcomes and were thereby able to assess the effectiveness of organisational interventions on mental health more precisely and with regard to the used measurement tools. In addition, including only controlled studies that used validated measurement tools increased the validity of our findings.

The focused attention for the inclusion of studies that were conducted with SMEs within healthcare is another strength of this review. However, it is also a limitation of this review that we did not identify studies conducted in independent SMEs, but only in SMEs that were attached to a larger organisational structure. We can therefore not draw any conclusion with regard to organisational interventions in healthcare SMEs. Our requirements for including studies might have been too strict (e.g. only controlled studies using validated measurement instruments) to include more SME studies as it seems that only few organisational intervention studies with this methodological quality are being conducted with SMEs so far. A search with less strict inclusion criteria and that includes grey literature might therefore be necessary to learn more about organisational interventions in SMEs. Nevertheless, the identified SME studies (all attached to larger organisational structures) were conducted in occupational groups, such as home care workers employed by the person receiving care, point at the need for innovative approaches that better protect and improve mental health and a greater representation and participation of SMEs in research studies.

Another limitation of this review is that it does not include a meta-analysis. However, due to our interest in assessing the effectiveness of all types of organisational interventions on a variety of outcomes, the identified studies were too heterogeneous for such an approach.

### Implications

Our review showed that mental health in health care workers can be improved through organisational interventions. Interventions that improve health care workers’ working environment through better work organization, more flexible working time arrangements or better professional competencies seem to be able to produce positive mental health outcomes, especially with regard to burnout. Workplaces should therefore investigate how the workflow can be improved, how employees can be supported with continued professional education and how to allow for better possibilities for restitution so that health care workers can conduct their important work in a healthy work environment. These approaches also need to be tested in SMEs, which tend to have fewer resources to create healthy workplaces for their employees.

## Conclusion

Organisational interventions in healthcare workers can be effective in improving mental health, especially in reducing burnout. The approach adopted in this review allowed for a detailed analysis of the effectiveness of specific intervention types and showed that participatory interventions, although potentially very effective, are often challenged by barriers to implementation, while interventions that consist of implemented workplace changes show a high level of evidence. A strong level of evidence was found for the intervention type “Job and task modifications” and a moderate level of evidence for the types “Flexible work and scheduling” and “Changes in the physical work environment”, illustrating that positive effects can be achieved through concrete changes at the workplace. More research is needed to determine which interventions work in healthcare SMEs, in non-hospital settings and with a wider range of occupations beyond physicians and nurses.

### Supplementary Information

Below is the link to the electronic supplementary material.

Supplementary file1 (PDF 521 KB) Appendix 1: Search string

Supplementary file (PDF 728 KB) Appendix 2: Detailed summary of quality appraisal results

Supplementary file1 (PDF 1034 KB) Appendix 3: Effectiveness of primary outcomes

Supplementary file1 (PDF 1114 KB) Appendix 4: Effectiveness of secondary outcomes

## Data Availability

This is a systematic review based on articles previously published in scientific journals. The search can be repeated by using the search string provided in Appendix 1. The inclusion and exclusion criteria are presented in Table 1. The references of the identified articles are provided in the article, as well as the detailed summary of the quality appraisal (Appendix 2). The approach for assessing the level of evidence is explained in the article and the results are shown in Table 5. More detailed presentations of the findings with regard to the primary and secondary outcomes that this review focused on are presented in Appendix 3 and 4.
