# Supplementary material for: The effects of different types of organisational workplace mental health interventions on mental health and wellbeing in healthcare workers: a systematic review
Source: Int Arch Occup Environ Health. 2024 May 2;97(5):485–522. doi: 10.1007/s00420-024-02065-z (PMC11130054; doi:10.1007/s00420-024-02065-z)
Supplement: Supplementary file 1 — Supplementary file1 (PDF 521 KB) Appendix 1: Search string [file 420_2024_2065_MOESM1_ESM.pdf]

PubMed Search (2020-07-03)

((("health personnel"[MeSH Terms] AND (("loattrfull text"[Filter] AND "humans"[MeSH Terms]) AND "english"[Language])) AND (((((((("randomized controlled trial"[Publication Type] OR "randomized controlled trials as topic"[MeSH Terms]) OR "random allocation"[MeSH Terms]) OR "double-blind method"[MeSH Terms]) OR "single-blind method"[MeSH Terms]) OR "random*"[Text Word]) OR "Placebos"[MeSH Terms]) OR "placebo"[Title/Abstract]) OR (((("singl*"[Text Word] OR "doubl*"[Text Word]) OR "trebl*"[Text Word]) OR "tripl*"[Text Word]) AND (("mask*"[Text Word] OR "blind*"[Text Word]) OR "dumm*"[Text Word]))) AND (("loattrfull text"[Filter] AND "humans"[MeSH Terms]) AND "english"[Language]))) AND ((((((((((("intervention*"[Title/Abstract] OR "program*"[Title/Abstract]) OR "prevention"[Title/Abstract]) OR "training"[Title/Abstract]) OR "initiative*"[Title/Abstract]) OR "policy"[Title/Abstract]) OR "promotion"[Title/Abstract]) OR "campaign*"[Title/Abstract]) AND (("loattrfull text"[Filter] AND "humans"[MeSH Terms]) AND "english"[Language])) OR ("primary prevention"[MeSH Terms] AND (("loattrfull text"[Filter] AND "humans"[MeSH Terms]) AND "english"[Language]))) OR ("secondary prevention"[MeSH Terms] AND (("loattrfull text"[Filter] AND "humans"[MeSH Terms]) AND "english"[Language]))) AND (("loattrfull text"[Filter] AND "humans"[MeSH Terms]) AND "english"[Language])) AND ((((("workplace"[MeSH Terms] AND (("loattrfull text"[Filter] AND "humans"[MeSH Terms]) AND "english"[Language])) OR ("organizations"[MeSH Terms] AND (("loattrfull text"[Filter] AND "humans"[MeSH Terms]) AND "english"[Language]))) OR (((((("worksite*"[Title/Abstract] OR "work site*"[Title/Abstract]) OR "job site*"[Title/Abstract]) OR "office*"[Title/Abstract]) OR "hospital*"[Title/Abstract]) OR "clinic*"[Title/Abstract]) AND (("loattrfull text"[Filter] AND "humans"[MeSH Terms]) AND "english"[Language]))) OR (("organization*"[Title/Abstract] OR "organisation*"[Title/Abstract]) AND (("loattrfull text"[Filter] AND "humans"[MeSH Terms]) AND "english"[Language]))) AND (("loattrfull text"[Filter] AND "humans"[MeSH Terms]) AND "english"[Language]))) AND (("loattrfull text"[Filter] AND "humans"[MeSH Terms]) AND "english"[Language]))) AND (((((((((((("depressive disorder"[MeSH Terms] AND (("loattrfull text"[Filter] AND "humans"[MeSH Terms]) AND "english"[Language])) OR ("depression"[MeSH Terms] AND (("loattrfull text"[Filter] AND "humans"[MeSH Terms]) AND "english"[Language]))) OR ("anxiety"[MeSH Terms] AND (("loattrfull text"[Filter] AND "humans"[MeSH Terms]) AND "english"[Language]))) OR ("burnout, professional"[MeSH Terms] AND (("loattrfull text"[Filter] AND "humans"[MeSH Terms]) AND "english"[Language]))) OR ("burnout, psychological"[MeSH Terms] AND (("loattrfull text"[Filter] AND "humans"[MeSH Terms]) AND "english"[Language]))) OR ("occupational stress"[MeSH Terms] AND (("loattrfull text"[Filter] AND "humans"[MeSH Terms]) AND "english"[Language]))) OR ((("well-being"[Title/Abstract] OR "well-being"[Title/Abstract]) OR "wellbeing"[Title/Abstract]) AND (("loattrfull text"[Filter] AND "humans"[MeSH Terms]) AND "english"[Language]))) OR ((("mental well being"[Title/Abstract] OR "mental well being"[Title/Abstract]) OR "mental wellbeing"[Title/Abstract]) AND (("loattrfull text"[Filter] AND "humans"[MeSH Terms]) AND "english"[Language]))) OR ("mental wellness"[Title/Abstract] AND (("loattrfull text"[Filter] AND "humans"[MeSH Terms]) AND "english"[Language]))) OR ("psychological health"[Title/Abstract] AND (("loattrfull text"[Filter] AND "humans"[MeSH Terms]) AND "english"[Language]))) OR ((("depressive symptoms"[Title/Abstract] OR "anxiety symptoms"[Title/Abstract]) OR "stress symptoms"[Title/Abstract]) AND (("loattrfull text"[Filter] AND "humans"[MeSH Terms]) AND "english"[Language]))) OR (("burnout"[Title/Abstract] OR "burn out"[Title/Abstract]) AND (("loattrfull text"[Filter] AND "humans"[MeSH Terms]) AND "english"[Language]))) OR ((("psychological wellbeing"[Title/Abstract] OR "psychological well being"[Title/Abstract]) OR "psychological well being"[Title/Abstract]) AND (("loattrfull text"[Filter] AND "humans"[MeSH Terms]) AND "english"[Language]))) AND (("loattrfull text"[Filter] AND "humans"[MeSH Terms]) AND "english"[Language]))

425 Results

#	Query	Limiters/Expanders	Last Run Via	Results
S37	S27 AND S34	Limiters - Published Date: 20100101-20201231<br>Expanders - Apply equivalent subjects<br>Narrow by Language: - english<br>Search modes - Boolean/Phrase	Interface - EBSCOhost Research Databases<br>Search Screen - Advanced Search<br>Database - Academic Search Complete	430
S36	S27 AND S34	Limiters - Published Date: 20100101-20201231<br>Expanders - Apply equivalent subjects<br>Search modes - Boolean/Phrase	Interface - EBSCOhost Research Databases<br>Search Screen - Advanced Search<br>Database - Academic Search Complete	448
S35	S27 AND S34	Expanders - Apply equivalent subjects<br>Search modes - Boolean/Phrase	Interface - EBSCOhost Research Databases<br>Search Screen - Advanced Search<br>Database - Academic Search Complete	552
S34	S28 OR S29 OR S30 OR S31 OR S32 OR S33	Expanders - Apply equivalent subjects<br>Search modes - Boolean/Phrase	Interface - EBSCOhost Research Databases<br>Search Screen - Advanced Search<br>Database - Academic Search Complete	1,207,095
S33	TI ((double-blind stud*) or (single-blind study*) or (random assignment) or (pretest-posttest design) or (cluster sample) or (placebo*) or (comparative stud*) or (control N5 group)) OR AB ((double-blind stud*) or (single-blind study*) or (random assignment) or (pretest-	Expanders - Apply equivalent subjects<br>Search modes - Boolean/Phrase	Interface - EBSCOhost Research Databases<br>Search Screen - Advanced Search<br>Database - Academic Search Complete	529,583

	posttest design) or (cluster sample) or (placebo*) or (comparative stud*) or (control N5 group))			
S32	AB random*	Expanders - Apply equivalent subjects<br>Search modes - Boolean/Phrase	Interface - EBSCOhost Research Databases<br>Search Screen - Advanced Search<br>Database - Academic Search Complete	803,943
S31	TI randomised or randomized	Expanders - Apply equivalent subjects<br>Search modes - Boolean/Phrase	Interface - EBSCOhost Research Databases<br>Search Screen - Advanced Search<br>Database - Academic Search Complete	139,449
S30	DE "BLIND experiment"	Expanders - Apply equivalent subjects<br>Search modes - Boolean/Phrase	Interface - EBSCOhost Research Databases<br>Search Screen - Advanced Search<br>Database - Academic Search Complete	13,268
S29	DE "CROSSOVER trials"	Expanders - Apply equivalent subjects<br>Search modes - Boolean/Phrase	Interface - EBSCOhost Research Databases<br>Search Screen - Advanced Search<br>Database - Academic Search Complete	5,768
S28	DE "RANDOMIZED controlled trials" OR DE "CLUSTER randomized controlled trials"	Expanders - Apply equivalent subjects<br>Search modes - Boolean/Phrase	Interface - EBSCOhost Research Databases<br>Search Screen - Advanced Search<br>Database - Academic Search Complete	90,377
S27	S6 AND S16 AND S26	Expanders - Apply equivalent subjects<br>Search modes - Boolean/Phrase	Interface - EBSCOhost Research Databases<br>Search Screen - Advanced Search<br>Database - Academic Search Complete	5,099
S26	S17 OR S18 OR S19 OR S20 OR S21 OR S22 OR	Expanders - Apply equivalent subjects	Interface - EBSCOhost Research Databases	480,134

	S23 OR S24 OR S25	Search modes - Boolean/Phrase	Search Screen - Advanced Search<br>Database - Academic Search Complete	
S25	TI (depressive symptoms or anxiety symptoms or stress symptoms or burnout) OR AB (depressive symptoms or anxiety symptoms or stress symptoms or burnout)	Expanders - Apply equivalent subjects<br>Search modes - Boolean/Phrase	Interface - EBSCOhost Research Databases<br>Search Screen - Advanced Search<br>Database - Academic Search Complete	87,771
S24	TI (psychological health or mental health) OR AB (psychological health or mental health)	Expanders - Apply equivalent subjects<br>Search modes - Boolean/Phrase	Interface - EBSCOhost Research Databases<br>Search Screen - Advanced Search<br>Database - Academic Search Complete	183,524
S23	TI (mental well-being or mental wellness or psychological well-being) OR AB (mental well-being or mental wellness or psychological well-being)	Expanders - Apply equivalent subjects<br>Search modes - Boolean/Phrase	Interface - EBSCOhost Research Databases<br>Search Screen - Advanced Search<br>Database - Academic Search Complete	16,766
S22	TI (wellbeing or wellbeing or well being) OR AB (wellbeing or wellbeing or well being)	Expanders - Apply equivalent subjects<br>Search modes - Boolean/Phrase	Interface - EBSCOhost Research Databases<br>Search Screen - Advanced Search<br>Database - Academic Search Complete	118,897
S21	SU psychological well-being	Expanders - Apply equivalent subjects<br>Search modes - Boolean/Phrase	Interface - EBSCOhost Research Databases<br>Search Screen - Advanced Search<br>Database - Academic Search Complete	3,313
S20	SU burnout	Expanders - Apply equivalent subjects<br>Search modes - Boolean/Phrase	Interface - EBSCOhost Research Databases<br>Search Screen - Advanced Search<br>Database - Academic Search Complete	7,290

S19	SU anxiety	Expanders - Apply equivalent subjects<br>Search modes - Boolean/Phrase	Interface - EBSCOhost Research Databases<br>Search Screen - Advanced Search<br>Database - Academic Search Complete	70,992
S18	SU mental depression	Expanders - Apply equivalent subjects<br>Search modes - Boolean/Phrase	Interface - EBSCOhost Research Databases<br>Search Screen - Advanced Search<br>Database - Academic Search Complete	107,143
S17	SU job stress	Expanders - Apply equivalent subjects<br>Search modes - Boolean/Phrase	Interface - EBSCOhost Research Databases<br>Search Screen - Advanced Search<br>Database - Academic Search Complete	12,282
S16	S10 AND S15	Expanders - Apply equivalent subjects<br>Search modes - Boolean/Phrase	Interface - EBSCOhost Research Databases<br>Search Screen - Advanced Search<br>Database - Academic Search Complete	655,791
S15	S11 OR S12 OR S13 OR S14	Expanders - Apply equivalent subjects<br>Search modes - Boolean/Phrase	Interface - EBSCOhost Research Databases<br>Search Screen - Advanced Search<br>Database - Academic Search Complete	3,516,837
S14	TI (worksite or work site or job site or office or clinic or hospital) OR AB (worksite or work site or job site or office or clinic or hospital)	Expanders - Apply equivalent subjects<br>Search modes - Boolean/Phrase	Interface - EBSCOhost Research Databases<br>Search Screen - Advanced Search<br>Database - Academic Search Complete	999,208
S13	TI (organization* or organisation*) OR AB (organization* or organisation*)	Expanders - Apply equivalent subjects<br>Search modes - Boolean/Phrase	Interface - EBSCOhost Research Databases<br>Search Screen - Advanced Search<br>Database - Academic Search Complete	671,678

S12	TI (work or workplace or work place) OR AB (work or workplace or work place)	Expanders - Apply equivalent subjects<br>Search modes - Boolean/Phrase	Interface - EBSCOhost Research Databases<br>Search Screen - Advanced Search<br>Database - Academic Search Complete	2,029,696
S11	SU work environment	Expanders - Apply equivalent subjects<br>Search modes - Boolean/Phrase	Interface - EBSCOhost Research Databases<br>Search Screen - Advanced Search<br>Database - Academic Search Complete	35,108
S10	S7 OR S8 OR S9	Expanders - Apply equivalent subjects<br>Search modes - Boolean/Phrase	Interface - EBSCOhost Research Databases<br>Search Screen - Advanced Search<br>Database - Academic Search Complete	3,553,612
S9	TI (intervention* or program* or prevention or training or initiative* or policy or promotion or campaign*) OR AB (intervention* or program* or prevention or training or initiative* or policy or promotion or campaign*)	Expanders - Apply equivalent subjects<br>Search modes - Boolean/Phrase	Interface - EBSCOhost Research Databases<br>Search Screen - Advanced Search<br>Database - Academic Search Complete	3,553,271
S8	SU mental health promotion	Expanders - Apply equivalent subjects<br>Search modes - Boolean/Phrase	Interface - EBSCOhost Research Databases<br>Search Screen - Advanced Search<br>Database - Academic Search Complete	604
S7	SU program implementation	Expanders - Apply equivalent subjects<br>Search modes - Boolean/Phrase	Interface - EBSCOhost Research Databases<br>Search Screen - Advanced Search<br>Database - Academic Search Complete	589
S6	S1 OR S2 OR S3 OR S4 OR S5	Expanders - Apply equivalent subjects<br>Search modes - Boolean/Phrase	Interface - EBSCOhost Research Databases<br>Search Screen - Advanced Search	334,679

			Database - Academic Search Complete	
S5	DE "MENTAL health personnel" OR DE "ALLIED mental health personnel" OR DE "CLINICAL psychologists" OR DE "COMMUNITY mental health personnel" OR DE "MENTAL health care teams" OR DE "MENTAL health clergy" OR DE "MENTAL health counselors" OR DE "MINORITY mental health personnel" OR DE "OLDER people in mental health" OR DE "PSYCHIATRIC nurses" OR DE "PSYCHIATRIC nursing" OR DE "PSYCHIATRIC social workers" OR DE "PSYCHIATRISTS" OR DE "PSYCHOTHERAPISTS" OR DE "STUDENT volunteers in mental health"	Expanders - Apply equivalent subjects<br>Search modes - Boolean/Phrase	Interface - EBSCOhost Research Databases<br>Search Screen - Advanced Search<br>Database - Academic Search Complete	23,455
S4	DE "PHYSICIANS" OR DE "ANESTHESIOLOGISTS" OR DE "CARDIOLOGISTS" OR DE "CHILDREN of physicians" OR DE "CHRISTIAN physicians" OR DE "EMERGENCY physicians" OR DE "FOREIGN physicians" OR DE "GERIATRICIANS" OR DE "GOVERNMENT physicians" OR DE "GYNECOLOGISTS" OR DE "HEMATOLOGISTS" OR DE "HOMEOPATHIC physicians" OR DE	Expanders - Apply equivalent subjects<br>Search modes - Boolean/Phrase	Interface - EBSCOhost Research Databases<br>Search Screen - Advanced Search<br>Database - Academic Search Complete	127,228

"HOSPITAL medical staff" OR DE "INDIGENOUS physicians" OR DE "INTERNISTS" OR DE "JEWISH physicians" OR DE "LGBTQ physicians" OR DE "MEDICAL consultants" OR DE "MEDICAL staff of public hospitals" OR DE "MEDICAL staff of veterans' hospitals" OR DE "MILITARY physicians" OR DE "MUSLIM physicians" OR DE "NATIVE American physicians" OR DE "NEPHROLOGISTS" OR DE "NEUROLOGISTS" OR DE "NUCLEAR medicine physicians" OR DE "NURSE-physician relationships" OR DE "OBSTETRICIANS" OR DE "OCCUPATIONAL physicians" OR DE "ONCOLOGISTS" OR DE "OPHTHALMOLOGISTS" OR DE "OSTEOPATHIC physicians" OR DE "OTOLARYNGOLOGISTS" OR DE "PATHOLOGISTS" OR DE "PEDIATRICIANS" OR DE "PHYSIATRISTS" OR DE "PHYSICIAN executives" OR DE "PHYSICIAN services utilization" OR DE "PHYSICIANS as authors" OR DE "PHYSICIANS as patients" OR DE "PHYSICIANS with disabilities" OR DE "PREVENTIVE medicine physicians" OR DE "PRISON physicians" OR DE "PSYCHIATRISTS" OR

	DE "PULMONOLOGISTS" OR DE "QUAKER physicians" OR DE "RADIOLOGISTS" OR DE "RESIDENTS (Medicine)" OR DE "SCHOOL physicians" OR DE "SHIP physicians" OR DE "SPORTS physicians" OR DE "STOMATOLOGISTS" OR DE "SUBSTITUTE physicians" OR DE "SURGEONS" OR DE "SURGEONS general (Military personnel)" OR DE "UROLOGISTS" OR DE "WOMEN physicians"			
S3	DE "NURSES" OR DE "ADVANCED practice registered nurses" OR DE "ASSOCIATE degree nurses" OR DE "COMMUNITY health nurses" OR DE "EXPATRIATE nurses" OR DE "FLOAT nurses" OR DE "GAY nurses" OR DE "HOSPICE nurses" OR DE "HOSPITAL nursing staff" OR DE "JEWISH nurses" OR DE "LGBTQ nurses" OR DE "MALE nurses" OR DE "NATIVE American nurses" OR DE "NEUROLOGICAL nurses" OR DE "NUNS as nurses" OR DE "NURSE administrators" OR DE "NURSE liaisons" OR DE "NURSE-physician relationships" OR DE "NURSES as patients" OR DE "NURSES with disabilities" OR DE "NURSES' aides" OR DE "NURSING consultants"	Expanders - Apply equivalent subjects<br>Search modes - Boolean/Phrase	Interface - EBSCOhost Research Databases<br>Search Screen - Advanced Search<br>Database - Academic Search Complete	72,345

	OR DE "NURSING students" OR DE "ONCOLOGY nurse navigators" OR DE "OPERATING room nurses" OR DE "PRACTICAL nurses" OR DE "PRISON nurses" OR DE "PSYCHIATRIC nurses" OR DE "PUBLIC health nurses" OR DE "RURAL nurses" OR DE "SEXUAL assault nurse examiners" OR DE "VISITING nurses"			
S2	DE "ALLIED health personnel" OR DE "ACUPUNCTURISTS" OR DE "ANESTHESIOLOGIST assistants" OR DE "ARTIFICIAL limb fitters" OR DE "ATHLETIC trainers" OR DE "AUDIOLOGISTS" OR DE "BIOMEDICAL technicians" OR DE "CARDIOVASCULAR technicians" OR DE "COMMUNITY health workers" OR DE "DENTAL auxiliary personnel" OR DE "DIETITIANS" OR DE "DOULAS" OR DE "ELECTROLOGISTS" OR DE "EMERGENCY medical technicians" OR DE "EXERCISE personnel" OR DE "HEALTH coaches" OR DE "HEALTH counselors" OR DE "HOME health aides" OR DE "HYPNOTHERAPISTS" OR DE "INDUSTRIAL hygienists" OR DE	Expanders - Apply equivalent subjects<br>Search modes - Boolean/Phrase	Interface - EBSCOhost Research Databases<br>Search Screen - Advanced Search<br>Database - Academic Search Complete	29,324

	"LACTATION consultants" OR DE "MEDICAL assistants" OR DE "MEDICAL laboratory assistants" OR DE "MEDICAL record personnel" OR DE "MEDICAL secretaries" OR DE "NURSES' aides" OR DE "NURSING home administrators" OR DE "OCCUPATIONAL therapists" OR DE "OCCUPATIONAL therapy assistants" OR DE "OPHTHALMIC assistants" OR DE "OPTICIANS" OR DE "OPTOMETRIC assistants" OR DE "ORIENTATION & mobility instructors" OR DE "PHYSICAL therapists" OR DE "PHYSICAL therapy assistants" OR DE "PHYSICIANS' assistants" OR DE "PODIATRIC assistants" OR DE "RECREATIONAL therapists" OR DE "RESPIRATORY therapists" OR DE "SPEECH therapists"			
S1	DE "MEDICAL personnel" OR DE "ABORIGINAL Australians in medicine" OR DE "ALLIED health personnel" OR DE "AROMATHERAPISTS" OR DE "BIOMEDICAL engineers" OR DE "BLACK people in medicine" OR DE "CHIROPRACTORS" OR DE "DENTAL personnel" OR DE "EMERGENCY medical personnel" OR DE	Expanders - Apply equivalent subjects<br>Search modes - Boolean/Phrase	Interface - EBSCOhost Research Databases<br>Search Screen - Advanced Search<br>Database - Academic Search Complete	228,254

"HEALTH care teams" OR DE "HEALTH occupations students" OR DE "HEALTH practitioners" OR DE "HEALTH services administrators" OR DE "HOSPITAL personnel" OR DE "IMPAIRED medical personnel" OR DE "MEDICAL personnel as patients" OR DE "MEDICAL personnel-caregiver relationships" OR DE "MEDICAL registry personnel" OR DE "MEDICAL research personnel" OR DE "MEDICAL scribes" OR DE "MEDICAL specialties and specialists" OR DE "MEDICAL teaching personnel" OR DE "MENTAL health personnel" OR DE "MIDWIVES" OR DE "MILITARY medical personnel" OR DE "MINORITY medical personnel" OR DE "MULTISKILLED medical personnel" OR DE "NATIVE Americans in medicine" OR DE "NURSES" OR DE "OPERATING room personnel" OR DE "OPTOMETRISTS" OR DE "ORGAN transplant coordinators" OR DE "PATIENT-professional relations" OR DE "PHARMACISTS" OR DE "PHLEBOTOMISTS" OR DE "PHYSICIANS" OR DE "PODIATRISTS" OR DE "PROSTHETISTS" OR DE

"PUBLIC health personnel" OR DE "RECOVERY room personnel" OR DE "STUDENT volunteers in medical care" OR DE "TRAVELING medical personnel" OR DE "UNLICENSED medical personnel"

Friday, July 03, 2020 11:54:33 AM

#	Query	Limiters/Expanders	Last Run Via	Results
S50	S23 AND S46	Limiters - Published Date: 20100101-20201231<br>Expanders - Apply equivalent subjects<br>Narrow by SubjectAge: - all adult<br>Narrow by Language: - english<br>Search modes - Boolean/Phrase	Interface - EBSCOhost Research Databases<br>Search Screen - Advanced Search<br>Database - CINAHL Plus with Full Text	558
S49	S23 AND S46	Limiters - Published Date: 20100101-20201231<br>Expanders - Apply equivalent subjects<br>Narrow by Language: - english<br>Search modes - Boolean/Phrase	Interface - EBSCOhost Research Databases<br>Search Screen - Advanced Search<br>Database - CINAHL Plus with Full Text	935
S48	S23 AND S46	Limiters - Published Date: 20100101-20201231<br>Expanders - Apply equivalent subjects<br>Search modes - Boolean/Phrase	Interface - EBSCOhost Research Databases<br>Search Screen - Advanced Search<br>Database - CINAHL Plus with Full Text	1,008
S47	S23 AND S46	Expanders - Apply equivalent subjects<br>Search modes - Boolean/Phrase	Interface - EBSCOhost Research Databases<br>Search Screen - Advanced Search<br>Database - CINAHL Plus with Full Text	1,416
S46	S45 NOT S44	Expanders - Apply equivalent subjects<br>Search modes - Boolean/Phrase	Interface - EBSCOhost Research Databases<br>Search Screen - Advanced Search<br>Database - CINAHL Plus with Full Text	788,959
S45	S24 OR S25 OR S26 OR	Expanders - Apply	Interface - EBSCOhost	829,094

	S27 OR S28 OR S29 OR S30 OR S31 OR S32 OR S33 OR S34 OR S35 OR S36 OR S37 OR S38	equivalent subjects Search modes - Boolean/Phrase	Research Databases Search Screen - Advanced Search Database - CINAHL Plus with Full Text	
S44	S42 NOT S43	Expanders - Apply equivalent subjects Search modes - Boolean/Phrase	Interface - EBSCOhost Research Databases Search Screen - Advanced Search Database - CINAHL Plus with Full Text	195,916
S43	MH (human)	Expanders - Apply equivalent subjects Search modes - Boolean/Phrase	Interface - EBSCOhost Research Databases Search Screen - Advanced Search Database - CINAHL Plus with Full Text	2,442,858
S42	S39 OR S40 OR S41	Expanders - Apply equivalent subjects Search modes - Boolean/Phrase	Interface - EBSCOhost Research Databases Search Screen - Advanced Search Database - CINAHL Plus with Full Text	224,570
S41	TI (animal model*)	Expanders - Apply equivalent subjects Search modes - Boolean/Phrase	Interface - EBSCOhost Research Databases Search Screen - Advanced Search Database - CINAHL Plus with Full Text	3,311
S40	MH ("animal studies")	Expanders - Apply equivalent subjects Search modes - Boolean/Phrase	Interface - EBSCOhost Research Databases Search Screen - Advanced Search Database - CINAHL Plus with Full Text	138,746
S39	MH ("animals+")	Expanders - Apply equivalent subjects Search modes - Boolean/Phrase	Interface - EBSCOhost Research Databases Search Screen - Advanced Search Database - CINAHL Plus with Full Text	94,072
S38	AB (cluster W3 RCT)	Expanders - Apply	Interface - EBSCOhost	378

		equivalent subjects<br>Search modes - Boolean/Phrase	Research Databases<br>Search Screen - Advanced Search<br>Database - CINAHL Plus with Full Text	
S37	MH ("crossover design") OR MH ("comparative studies")	Expanders - Apply equivalent subjects<br>Search modes - Boolean/Phrase	Interface - EBSCOhost Research Databases<br>Search Screen - Advanced Search<br>Database - CINAHL Plus with Full Text	359,651
S36	AB (control W5 group)	Expanders - Apply equivalent subjects<br>Search modes - Boolean/Phrase	Interface - EBSCOhost Research Databases<br>Search Screen - Advanced Search<br>Database - CINAHL Plus with Full Text	121,427
S35	PT randomized controlled trial	Expanders - Apply equivalent subjects<br>Search modes - Boolean/Phrase	Interface - EBSCOhost Research Databases<br>Search Screen - Advanced Search<br>Database - CINAHL Plus with Full Text	133,026
S34	MH ("placebos")	Expanders - Apply equivalent subjects<br>Search modes - Boolean/Phrase	Interface - EBSCOhost Research Databases<br>Search Screen - Advanced Search<br>Database - CINAHL Plus with Full Text	13,771
S33	MH ("sample size") AND AB (assigned or allocated or control)	Expanders - Apply equivalent subjects<br>Search modes - Boolean/Phrase	Interface - EBSCOhost Research Databases<br>Search Screen - Advanced Search<br>Database - CINAHL Plus with Full Text	4,865
S32	TI trial	Expanders - Apply equivalent subjects<br>Search modes - Boolean/Phrase	Interface - EBSCOhost Research Databases<br>Search Screen - Advanced Search<br>Database - CINAHL Plus with Full Text	113,857
S31	AB random*	Expanders - Apply	Interface - EBSCOhost	337,345

		equivalent subjects<br>Search modes - Boolean/Phrase	Research Databases<br>Search Screen - Advanced Search<br>Database - CINAHL Plus with Full Text	
S30	TI randomised or randomized	Expanders - Apply equivalent subjects<br>Search modes - Boolean/Phrase	Interface - EBSCOhost Research Databases<br>Search Screen - Advanced Search<br>Database - CINAHL Plus with Full Text	111,933
S29	MH ("cluster sample")	Expanders - Apply equivalent subjects<br>Search modes - Boolean/Phrase	Interface - EBSCOhost Research Databases<br>Search Screen - Advanced Search<br>Database - CINAHL Plus with Full Text	5,285
S28	MH ("pretest-posttest design")	Expanders - Apply equivalent subjects<br>Search modes - Boolean/Phrase	Interface - EBSCOhost Research Databases<br>Search Screen - Advanced Search<br>Database - CINAHL Plus with Full Text	51,356
S27	MH ("random assignment")	Expanders - Apply equivalent subjects<br>Search modes - Boolean/Phrase	Interface - EBSCOhost Research Databases<br>Search Screen - Advanced Search<br>Database - CINAHL Plus with Full Text	68,776
S26	MH ("single-blind studies")	Expanders - Apply equivalent subjects<br>Search modes - Boolean/Phrase	Interface - EBSCOhost Research Databases<br>Search Screen - Advanced Search<br>Database - CINAHL Plus with Full Text	15,423
S25	MH ("double-blind studies")	Expanders - Apply equivalent subjects<br>Search modes - Boolean/Phrase	Interface - EBSCOhost Research Databases<br>Search Screen - Advanced Search<br>Database - CINAHL Plus with Full Text	50,446
S24	MH ("randomized	Expanders - Apply	Interface - EBSCOhost	120,878

	controlled trials")	equivalent subjects<br>Search modes - Boolean/Phrase	Research Databases<br>Search Screen - Advanced Search<br>Database - CINAHL Plus with Full Text	
S23	S1 AND S12 AND S22	Expanders - Apply equivalent subjects<br>Search modes - Boolean/Phrase	Interface - EBSCOhost Research Databases<br>Search Screen - Advanced Search<br>Database - CINAHL Plus with Full Text	10,098
S22	S13 OR S14 OR S15 OR S16 OR S17 OR S18 OR S19 OR S20 OR S21	Expanders - Apply equivalent subjects<br>Search modes - Boolean/Phrase	Interface - EBSCOhost Research Databases<br>Search Screen - Advanced Search<br>Database - CINAHL Plus with Full Text	296,567
S21	TI (burnout or burn-out or burn out) OR AB (burnout or burn-out or burn out)	Expanders - Apply equivalent subjects<br>Search modes - Boolean/Phrase	Interface - EBSCOhost Research Databases<br>Search Screen - Advanced Search<br>Database - CINAHL Plus with Full Text	10,640
S20	TI (depressive symptoms or anxiety symptoms or stress symptoms) OR AB (depressive symptoms or anxiety symptoms or stress symptoms)	Expanders - Apply equivalent subjects<br>Search modes - Boolean/Phrase	Interface - EBSCOhost Research Databases<br>Search Screen - Advanced Search<br>Database - CINAHL Plus with Full Text	46,496
S19	TI (psychological health or mental health) OR AB (psychological health or mental health)	Expanders - Apply equivalent subjects<br>Search modes - Boolean/Phrase	Interface - EBSCOhost Research Databases<br>Search Screen - Advanced Search<br>Database - CINAHL Plus with Full Text	127,696
S18	TI (mental well-being or mental wellness or psychological well-being) OR AB (mental well-being or mental wellness or psychological well-being)	Expanders - Apply equivalent subjects<br>Search modes - Boolean/Phrase	Interface - EBSCOhost Research Databases<br>Search Screen - Advanced Search<br>Database - CINAHL Plus with Full Text	10,672

S17	(MM "Psychological Well-being")	Expanders - Apply equivalent subjects<br>Search modes - Boolean/Phrase	Interface - EBSCOhost Research Databases<br>Search Screen - Advanced Search<br>Database - CINAHL Plus with Full Text	13,282
S16	(MM "Mental Health")	Expanders - Apply equivalent subjects<br>Search modes - Boolean/Phrase	Interface - EBSCOhost Research Databases<br>Search Screen - Advanced Search<br>Database - CINAHL Plus with Full Text	23,330
S15	(MM "Stress") OR (MM "Stress, Psychological") OR (MM "Compassion Fatigue") OR (MM "Critical Incident Stress") OR (MH "Stress, Occupational+") OR (MM "Role Stress")	Expanders - Apply equivalent subjects<br>Search modes - Boolean/Phrase	Interface - EBSCOhost Research Databases<br>Search Screen - Advanced Search<br>Database - CINAHL Plus with Full Text	63,887
S14	MM ("Anxiety")	Expanders - Apply equivalent subjects<br>Search modes - Boolean/Phrase	Interface - EBSCOhost Research Databases<br>Search Screen - Advanced Search<br>Database - CINAHL Plus with Full Text	21,044
S13	MM ("Depression")	Expanders - Apply equivalent subjects<br>Search modes - Boolean/Phrase	Interface - EBSCOhost Research Databases<br>Search Screen - Advanced Search<br>Database - CINAHL Plus with Full Text	69,540
S12	S6 AND S11	Expanders - Apply equivalent subjects<br>Search modes - Boolean/Phrase	Interface - EBSCOhost Research Databases<br>Search Screen - Advanced Search<br>Database - CINAHL Plus with Full Text	322,441
S11	S7 OR S8 OR S9 OR S10	Expanders - Apply equivalent subjects<br>Search modes - Boolean/Phrase	Interface - EBSCOhost Research Databases<br>Search Screen - Advanced Search	882,908

			Database - CINAHL Plus with Full Text	
S10	MM ("work environment")	Expanders - Apply equivalent subjects<br>Search modes - Boolean/Phrase	Interface - EBSCOhost Research Databases<br>Search Screen - Advanced Search<br>Database - CINAHL Plus with Full Text	16,017
S9	TI (worksite or work site or job site or office or hospital or clinic) OR AB (worksite or work site or job site or office or hospital or clinic)	Expanders - Apply equivalent subjects<br>Search modes - Boolean/Phrase	Interface - EBSCOhost Research Databases<br>Search Screen - Advanced Search<br>Database - CINAHL Plus with Full Text	528,146
S8	TI (organization or organisation or organizational or organisational) OR AB (organization or organisation or organizational or organisational)	Expanders - Apply equivalent subjects<br>Search modes - Boolean/Phrase	Interface - EBSCOhost Research Databases<br>Search Screen - Advanced Search<br>Database - CINAHL Plus with Full Text	151,405
S7	TI (work or workplace or work place) OR AB (work or workplace or work place)	Expanders - Apply equivalent subjects<br>Search modes - Boolean/Phrase	Interface - EBSCOhost Research Databases<br>Search Screen - Advanced Search<br>Database - CINAHL Plus with Full Text	289,386
S6	S2 OR S3 OR S4 OR S5	Expanders - Apply equivalent subjects<br>Search modes - Boolean/Phrase	Interface - EBSCOhost Research Databases<br>Search Screen - Advanced Search<br>Database - CINAHL Plus with Full Text	1,304,568
S5	TI (intervention* or program* or prevention or training or initiative* or policy or promotion or campaign*) OR AB (intervention* or program* or prevention or training or initiative* or policy or promotion or campaign*)	Expanders - Apply equivalent subjects<br>Search modes - Boolean/Phrase	Interface - EBSCOhost Research Databases<br>Search Screen - Advanced Search<br>Database - CINAHL Plus with Full Text	1,186,550

S4	MH ("Psychology, Occupational+")	Expanders - Apply equivalent subjects<br>Search modes - Boolean/Phrase	Interface - EBSCOhost Research Databases<br>Search Screen - Advanced Search<br>Database - CINAHL Plus with Full Text	109,524
S3	MH ("Program Evaluation")	Expanders - Apply equivalent subjects<br>Search modes - Boolean/Phrase	Interface - EBSCOhost Research Databases<br>Search Screen - Advanced Search<br>Database - CINAHL Plus with Full Text	44,290
S2	MH ("Program Development+")	Expanders - Apply equivalent subjects<br>Search modes - Boolean/Phrase	Interface - EBSCOhost Research Databases<br>Search Screen - Advanced Search<br>Database - CINAHL Plus with Full Text	89,657
S1	MH ("Health Personnel+")	Expanders - Apply equivalent subjects<br>Search modes - Boolean/Phrase	Interface - EBSCOhost Research Databases<br>Search Screen - Advanced Search<br>Database - CINAHL Plus with Full Text	603,730

MY EBSCOhost

Friday, July 03, 2020 2:17:32 PM

#	Query	Limiters/Expanders	Last Run Via	Results
S42	S33 AND S39	Limiters - Publication Year: 2010-2020<br>Expanders - Apply equivalent subjects<br>Search modes - Boolean/Phrase	Interface - EBSCOhost Research Databases<br>Search Screen - Advanced Search<br>Database - APA PsycInfo	333
S41	S33 AND S39	Limiters - Publication Year: 2010-2020<br>Expanders - Apply equivalent subjects<br>Search modes - Boolean/Phrase	Interface - EBSCOhost Research Databases<br>Search Screen - Advanced Search<br>Database - APA PsycInfo	333
S40	S33 AND S39	Expanders - Apply equivalent subjects<br>Search modes - Boolean/Phrase	Interface - EBSCOhost Research Databases<br>Search Screen - Advanced Search<br>Database - APA PsycInfo	517
S39	S34 OR S35 OR S36 OR S37 OR S38	Expanders - Apply equivalent subjects<br>Search modes - Boolean/Phrase	Interface - EBSCOhost Research Databases<br>Search Screen - Advanced Search<br>Database - APA PsycInfo	325,789
S38	TI ((double-blind stud*) or (single-blind study*) or (random assignment) or (pretest-posttest design) or (cluster sample) or (placebo*) or (comparative stud*) or (control N5 group)) OR AB ((double-blind stud*) or (single-blind study*) or (random assignment) or (pretest-posttest design) or (cluster sample) or (placebo*) or	Expanders - Apply equivalent subjects<br>Search modes - Boolean/Phrase	Interface - EBSCOhost Research Databases<br>Search Screen - Advanced Search<br>Database - APA PsycInfo	173,735

	(comparative stud*) or (control N5 group))			
S37	AB random*	Expanders - Apply equivalent subjects<br>Search modes - Boolean/Phrase	Interface - EBSCOhost Research Databases<br>Search Screen - Advanced Search<br>Database - APA PsycInfo	197,741
S36	TI (randomized) or (randomised)	Expanders - Apply equivalent subjects<br>Search modes - Boolean/Phrase	Interface - EBSCOhost Research Databases<br>Search Screen - Advanced Search<br>Database - APA PsycInfo	26,459
S35	MM "Random Sampling"	Expanders - Apply equivalent subjects<br>Search modes - Boolean/Phrase	Interface - EBSCOhost Research Databases<br>Search Screen - Advanced Search<br>Database - APA PsycInfo	583
S34	DE "Randomized Controlled Trials" OR DE "Randomized Clinical Trials"	Expanders - Apply equivalent subjects<br>Search modes - Boolean/Phrase	Interface - EBSCOhost Research Databases<br>Search Screen - Advanced Search<br>Database - APA PsycInfo	607
S33	S6 AND S20 AND S32	Expanders - Apply equivalent subjects<br>Search modes - Boolean/Phrase	Interface - EBSCOhost Research Databases<br>Search Screen - Advanced Search<br>Database - APA PsycInfo	6,200
S32	S21 OR S22 OR S23 OR S24 OR S25 OR S26 OR S27 OR S28 OR S29 OR S30 OR S31	Expanders - Apply equivalent subjects<br>Search modes - Boolean/Phrase	Interface - EBSCOhost Research Databases<br>Search Screen - Advanced Search<br>Database - APA PsycInfo	464,808
S31	TI (burnout or burn-out or burn out) OR AB (burnout or burn-out or burn out)	Expanders - Apply equivalent subjects<br>Search modes - Boolean/Phrase	Interface - EBSCOhost Research Databases<br>Search Screen - Advanced Search<br>Database - APA PsycInfo	12,426
S30	TI (depressive symptoms or anxiety symptoms or stress symptoms) OR AB (depressive symptoms or	Expanders - Apply equivalent subjects<br>Search modes - Boolean/Phrase	Interface - EBSCOhost Research Databases<br>Search Screen - Advanced Search<br>Database - APA PsycInfo	81,047

	anxiety symptoms or stress symptoms)			
S29	TI (psychological health or mental health) OR AB (psychological health or mental health)	Expanders - Apply equivalent subjects<br>Search modes - Boolean/Phrase	Interface - EBSCOhost Research Databases<br>Search Screen - Advanced Search<br>Database - APA PsycInfo	195,498
S28	TI (mental well-being or mental wellness or psychological well-being) OR AB (mental well-being or mental wellness or psychological well-being)	Expanders - Apply equivalent subjects<br>Search modes - Boolean/Phrase	Interface - EBSCOhost Research Databases<br>Search Screen - Advanced Search<br>Database - APA PsycInfo	19,436
S27	DE well being	Expanders - Apply equivalent subjects<br>Search modes - Boolean/Phrase	Interface - EBSCOhost Research Databases<br>Search Screen - Advanced Search<br>Database - APA PsycInfo	43,613
S26	DE mental health	Expanders - Apply equivalent subjects<br>Search modes - Boolean/Phrase	Interface - EBSCOhost Research Databases<br>Search Screen - Advanced Search<br>Database - APA PsycInfo	72,065
S25	DE Positive psychology	Expanders - Apply equivalent subjects<br>Search modes - Boolean/Phrase	Interface - EBSCOhost Research Databases<br>Search Screen - Advanced Search<br>Database - APA PsycInfo	4,704
S24	DE Occupational Stress	Expanders - Apply equivalent subjects<br>Search modes - Boolean/Phrase	Interface - EBSCOhost Research Databases<br>Search Screen - Advanced Search<br>Database - APA PsycInfo	21,450
S23	DE Stress	Expanders - Apply equivalent subjects<br>Search modes - Boolean/Phrase	Interface - EBSCOhost Research Databases<br>Search Screen - Advanced Search<br>Database - APA PsycInfo	61,184
S22	DE Anxiety	Expanders - Apply equivalent subjects	Interface - EBSCOhost Research Databases<br>Search Screen - Advanced	79,878

		Search modes - Boolean/Phrase	Search<br>Database - APA PsycInfo	
S21	DE Depression	Expanders - Apply equivalent subjects<br>Search modes - Boolean/Phrase	Interface - EBSCOhost Research Databases<br>Search Screen - Advanced Search<br>Database - APA PsycInfo	45,310
S20	S15 AND S19	Expanders - Apply equivalent subjects<br>Search modes - Boolean/Phrase	Interface - EBSCOhost Research Databases<br>Search Screen - Advanced Search<br>Database - APA PsycInfo	266,463
S19	S16 OR S17 OR S18	Expanders - Apply equivalent subjects<br>Search modes - Boolean/Phrase	Interface - EBSCOhost Research Databases<br>Search Screen - Advanced Search<br>Database - APA PsycInfo	875,804
S18	TI (worksite or work site or job site or office or clinic or hospital) OR AB (worksite or work site or job site or office or clinic or hospital)	Expanders - Apply equivalent subjects<br>Search modes - Boolean/Phrase	Interface - EBSCOhost Research Databases<br>Search Screen - Advanced Search<br>Database - APA PsycInfo	200,006
S17	TI ((organization or organisation or organizational or organisational)) OR AB ((organization or organisation or organizational or organisational))	Expanders - Apply equivalent subjects<br>Search modes - Boolean/Phrase	Interface - EBSCOhost Research Databases<br>Search Screen - Advanced Search<br>Database - APA PsycInfo	261,282
S16	TI (work or workplace or work place) OR AB (work or workplace or work place)	Expanders - Apply equivalent subjects<br>Search modes - Boolean/Phrase	Interface - EBSCOhost Research Databases<br>Search Screen - Advanced Search<br>Database - APA PsycInfo	521,503
S15	S7 OR S8 OR S9 OR S10 OR S11 OR S12 OR S13 OR S14	Expanders - Apply equivalent subjects<br>Search modes - Boolean/Phrase	Interface - EBSCOhost Research Databases<br>Search Screen - Advanced Search<br>Database - APA PsycInfo	1,056,832
S14	TI ((intervention* or	Expanders - Apply	Interface - EBSCOhost	1,047,743

	program* or prevention or training or initiative* or policy or promotion or campaign*)) OR AB ((intervention* or program* or prevention or training or initiative* or policy or promotion or campaign*))	equivalent subjects<br>Search modes - Boolean/Phrase	Research Databases<br>Search Screen - Advanced Search<br>Database - APA PsycInfo	
S13	DE primary mental health prevention	Expanders - Apply equivalent subjects<br>Search modes - Boolean/Phrase	Interface - EBSCOhost Research Databases<br>Search Screen - Advanced Search<br>Database - APA PsycInfo	2,385
S12	DE occupational health psychology	Expanders - Apply equivalent subjects<br>Search modes - Boolean/Phrase	Interface - EBSCOhost Research Databases<br>Search Screen - Advanced Search<br>Database - APA PsycInfo	155
S11	DE prevention	Expanders - Apply equivalent subjects<br>Search modes - Boolean/Phrase	Interface - EBSCOhost Research Databases<br>Search Screen - Advanced Search<br>Database - APA PsycInfo	30,518
S10	DE mental health program evaluation	Expanders - Apply equivalent subjects<br>Search modes - Boolean/Phrase	Interface - EBSCOhost Research Databases<br>Search Screen - Advanced Search<br>Database - APA PsycInfo	2,134
S9	DE program evaluation	Expanders - Apply equivalent subjects<br>Search modes - Boolean/Phrase	Interface - EBSCOhost Research Databases<br>Search Screen - Advanced Search<br>Database - APA PsycInfo	23,503
S8	DE workplace intervention	Expanders - Apply equivalent subjects<br>Search modes - Boolean/Phrase	Interface - EBSCOhost Research Databases<br>Search Screen - Advanced Search<br>Database - APA PsycInfo	699
S7	DE intervention	Expanders - Apply equivalent subjects<br>Search modes - Boolean/Phrase	Interface - EBSCOhost Research Databases<br>Search Screen - Advanced	64,874

			Search<br>Database - APA PsycInfo	
S6	S1 OR S2 OR S3 OR S4 OR S5	Expanders - Apply equivalent subjects<br>Search modes - Boolean/Phrase	Interface - EBSCOhost Research Databases<br>Search Screen - Advanced Search<br>Database - APA PsycInfo	151,287
S5	DE "Mental Health Personnel" OR DE "Clinical Psychologists" OR DE "Psychiatric Hospital Staff" OR DE "Psychiatric Nurses" OR DE "Psychiatric Social Workers" OR DE "Psychiatrists" OR DE "Psychotherapists" OR DE "School Psychologists"	Expanders - Apply equivalent subjects<br>Search modes - Boolean/Phrase	Interface - EBSCOhost Research Databases<br>Search Screen - Advanced Search<br>Database - APA PsycInfo	41,175
S4	DE "Medical Personnel" OR DE "Dentists" OR DE "Military Medical Personnel" OR DE "Nurses" OR DE "Optometrists" OR DE "Pharmacists" OR DE "Physical Therapists" OR DE "Physicians" OR DE "Psychiatric Hospital Staff"	Expanders - Apply equivalent subjects<br>Search modes - Boolean/Phrase	Interface - EBSCOhost Research Databases<br>Search Screen - Advanced Search<br>Database - APA PsycInfo	58,603
S3	DE "Medical Personnel" OR DE "Dentists" OR DE "Military Medical Personnel" OR DE "Nurses" OR DE "Optometrists" OR DE "Pharmacists" OR DE "Physical Therapists" OR DE "Physicians" OR DE "Psychiatric Hospital Staff"	Expanders - Apply equivalent subjects<br>Search modes - Boolean/Phrase	Interface - EBSCOhost Research Databases<br>Search Screen - Advanced Search<br>Database - APA PsycInfo	58,603
S2	DE "Allied Health Personnel" OR DE "Home	Expanders - Apply equivalent subjects	Interface - EBSCOhost Research Databases	6,483

	Care Personnel" OR DE "Occupational Therapists" OR DE "Physical Therapists" OR DE "Psychiatric Aides" OR DE "Speech Therapists"	Search modes - Boolean/Phrase	Search Screen - Advanced Search<br>Database - APA PsycInfo	
S1	DE "Health Personnel" OR DE "Allied Health Personnel" OR DE "Caregivers" OR DE "Medical Personnel" OR DE "Mental Health Personnel"	Expanders - Apply equivalent subjects<br>Search modes - Boolean/Phrase	Interface - EBSCOhost Research Databases<br>Search Screen - Advanced Search<br>Database - APA PsycInfo	71,300
