# Supplementary material for: The effects of different types of organisational workplace mental health interventions on mental health and wellbeing in healthcare workers: a systematic review
Source: Int Arch Occup Environ Health. 2024 May 2;97(5):485–522. doi: 10.1007/s00420-024-02065-z (PMC11130054; doi:10.1007/s00420-024-02065-z)
Supplement: Supplementary file 2 — Supplementary file (PDF 728 KB) Appendix 2: Detailed summary of quality appraisal results [file 420_2024_2065_MOESM2_ESM.pdf]

Appendix 2: Detailed summary of quality appraisal results

First Author, Year	Selection Bias	Design	Confounders	Blinding	Data Collection Method	Withdrawals and Drop-out	Global Rating
**Ali, 2011**	Weak	Moderate	Moderate	Moderate	Moderate	Moderate	**Moderate**
**Barcons, 2019**	Strong	Moderate	Moderate	Weak	Strong	Strong	**Moderate**
**Bourbonnais, 2011**	Moderate	Moderate	Strong	Moderate	Strong	Weak	**Moderate**
**Cordoza, 2018**	Weak	Weak	Weak	Strong	Moderate	Moderate	**Weak**
**Deneckere, 2013**	Moderate	Strong	Strong	Weak	Strong	Moderate	**Moderate**
**Emani, 2020**	Moderate	Strong	Strong	Weak	Strong	Moderate	**Moderate**
**Garland, 2012**	Moderate	Moderate	Strong	Moderate	Strong	Moderate	**Strong**
**Gregory, 2018**	Moderate	Moderate	Weak	Weak	Strong	Weak	**Weak**
**Havermans, 2018**	Moderate	Moderate	Strong	Moderate	Strong	Moderate	**Moderate**
**Jakobsen, 2017**	Moderate	Strong	Strong	Moderate	Moderate	Moderate	**Strong**
**Kossek, 2019**	Weak	Strong	Weak	Strong	Strong	Weak	**Weak**
**Leiter, 2011**	Weak	Moderate	Weak	Weak	Strong	Weak	**Weak**
**Linzer, 2015**	Moderate	Strong	Strong	Moderate	Moderate	Moderate	**Strong**
**Olson, 2016**	Moderate	Strong	Moderate	Weak	Moderate	Strong	**Moderate**
**Redhead, 2011**	Moderate	Moderate	Strong	Weak	Moderate	Strong	**Moderate**
**Saffari, 2021**	Strong	Strong	Strong	Strong	Strong	Strong	**Strong**
**Stansfeld, 2015**	Moderate	Strong	Moderate	Moderate	Strong	Moderate	**Strong**
**Tran, 2010**	Strong	Moderate	Strong	Weak	Strong	Weak	**Weak**
**Uchiyama, 2013**	Strong	Strong	Strong	Moderate	Strong	Strong	**Strong**
**West, 2014**	Moderate	Strong	Moderate	Moderate	Strong	Strong	**Strong**
**White, 2010**	Weak	Strong	Strong	Weak	Moderate	Weak	**Weak**
**Van Woerkom, 2021**	Moderate	Moderate	Strong	Moderate	Strong	Weak	**Moderate**

Appendix 2: Detailed summary of quality appraisal results (with color-coding)

FIRST AUTHOR, YEAR	SELECTION BIAS	DESIGN	CONFOUNDERS	BLINDING	DATA COLLECTION METHOD	WITHDRAWALS AND DROP-OUT	GLOBAL RATING
ALI, 2011	Weak	Moderate	Moderate	Moderate	Moderate	Moderate	Moderate
BARCONS, 2019	Strong	Moderate	Moderate	Weak	Strong	Strong	Moderate
BOURBONNAIS, 2011	Moderate	Moderate	Strong	Moderate	Strong	Weak	Moderate
CORDOZA, 2018	Weak	Weak	Weak	Strong	Moderate	Moderate	Weak
DENECKERE, 2013	Moderate	Strong	Strong	Weak	Strong	Moderate	Moderate
EMANI, 2020	Moderate	Strong	Strong	Weak	Strong	Moderate	Moderate
GARLAND, 2012	Moderate	Moderate	Strong	Moderate	Strong	Moderate	Strong
GREGORY, 2018	Moderate	Moderate	Weak	Weak	Strong	Weak	Weak
HAVERMANS, 2018	Moderate	Moderate	Strong	Moderate	Strong	Moderate	Moderate
JAKOBSEN, 2017	Moderate	Strong	Strong	Moderate	Moderate	Moderate	Strong
KOSSEK, 2019	Weak	Strong	Weak	Strong	Strong	Weak	Weak
LEITER, 2011	Weak	Moderate	Weak	Weak	Strong	Weak	Weak
LINZER, 2015	Moderate	Strong	Strong	Moderate	Moderate	Moderate	Strong
OLSON, 2016	Moderate	Strong	Moderate	Weak	Moderate	Strong	Moderate
REDHEAD, 2011	Moderate	Moderate	Strong	Weak	Moderate	Strong	Moderate
SAFFARI, 2021	Strong	Strong	Strong	Strong	Strong	Strong	Strong
STANSFELD, 2015	Moderate	Strong	Moderate	Moderate	Strong	Moderate	Strong
TRAN, 2010	Strong	Moderate	Strong	Weak	Strong	Weak	Weak
UCHIYAMA, 2013	Strong	Strong	Strong	Moderate	Strong	Strong	Strong
WEST, 2014	Moderate	Strong	Moderate	Moderate	Strong	Strong	Strong
WHITE, 2010	Weak	Strong	Strong	Weak	Moderate	Weak	Weak
VAN WOERKOM, 2021	Moderate	Moderate	Strong	Moderate	Strong	Weak	Moderate
