# Supplementary material for: The effects of different types of organisational workplace mental health interventions on mental health and wellbeing in healthcare workers: a systematic review
Source: Int Arch Occup Environ Health. 2024 May 2;97(5):485–522. doi: 10.1007/s00420-024-02065-z (PMC11130054; doi:10.1007/s00420-024-02065-z)
Supplement: Supplementary file 3 — Supplementary file1 (PDF 1034 KB) Appendix 3: Effectiveness of primary outcomes [file 420_2024_2065_MOESM3_ESM.pdf]

Appendix 3: Effectiveness of primary outcomes

First Author, Year	Outcome /Measurement	Scale (Scale Range)	Intervention Group(s)			Control Group			Effect (95% CI)
			N (N Post)	Pre-intervention Mean (SD)	Post-intervention Mean (SD)	N (N Post)	Pre-intervention Mean (SD)	Post-intervention Mean (SD)	
Ali, 2011	Burnout	Fenwick & Taussig, 2001(5-25)	Not reported	Not reported	12.35 (4.54)	Not reported	Not reported	15.40 (4.46)	Adjusted mean difference: 2.77 (0.97 to 4.58), p=.003* (adjusted for physician clustering, study site and workload)
	Stress	Fenwick & Taussig, 2001(6-30)		Not reported	13.36 (3.40)	Not reported	Not reported	14.64 (4.09)	Adjusted mean difference: 1.52 (0.83 to 2.22), p< .001* (adjusted for physician clustering, study site and workload)
Barcons, 2019	Mental Wellbeing	Brief Psychiatric Rating Scale (18-126)	20	23.50 (22 to 24.25)	20.50 (19 to 22)	18	25.50 (23.25 to 27.75)	23.50 (21 to 26)	Changes pre-post in intervention group significant (p=.001), but not control group, (p=.122) nor overall or intergroup evolution differences, p=.147
	Burnout	MBI EE 0-54, DP (0-30) SE (0-48)	20	EE=19.50 (13.75 to 23.25); DP=6 (4 to 8.50); PA=38 (34 to 42.50); Overall burnout=37 (29 to 49.25)	EE=21.50 (15.5 to 28.25); DP=7 (3.75 to 12.25); PA=37.50 (29.75 to 43); Overall burnout=40 (33 to 52)	18	EE=24.50 (14 to 41.25); DP=7 (5.25 to 13.75); PA=38 (33.25 to 45.25); Overall burnout=46.50 (24.50 to 62.25)	EE=25.50 (18 to 34.25); DP=9 (7.25 to 11); PA=38 (32.25 to 43.75); Overall burnout=46 (30 to 57.25)	Inter-group evolution differences: EE p=.263; DP p=.807; PA p=.550; Overall burnout p=.637

Appendix 3: Effectiveness of primary outcomes

First Author, Year	Outcome /Measurement	Scale (Scale Range)	Intervention Group(s)			Control Group			Effect (95% CI)
			N (N Post)	Pre-intervention Mean (SD)	Post-intervention Mean (SD)	N (N Post)	Pre-intervention Mean (SD)	Post-intervention Mean (SD)	
Bourbonnais, 2011	Stress	Abridged version (14 items) of the Psychiatric Symptom Index, PSI (0-100)	492 (248)	Mean (95% CI), SD 21.9 (20.6 to 23.2), 16.4	Mean (95% CI) at 12 months adjusted for baseline 20.4 (18.9 to 22.0)	618 (240)	Mean (95% CI) 21.6 (20.3-22.9) SD 14.9	Mean (95% CI) at 12 months adjusted for M0 22.4 (20.8 to 23.9)	Means at M2 adjusted for baseline p=.083
	Burnout	Copenhagen Burnout Inventory, CBI)		Mean (95% CI)	Mean (95% CI) at 12 months adjusted for baseline		Mean (95% CI)	Mean (95% CI) at 12 months adjusted for baseline	
		Client-related burnout (0-60)		34.9 (33.5 to 36.3)	33.4 (31.7 to 35.2)		36.3 (34.9 to 37.7)	37.4 (35.5 to 39.2)	Client-related burnout p=.003*
		Work-related burnout (0-70)		48.2 (46.8 to 49.5)	43.2 (41.5 to 44.9)		48.1 (46.7 to 49.5)	48.3 (46.6 to 50.1)	Work-related burnout p<0.0001*
		Personal burnout (0-60)		43.3 (41.9 to 44.7),	40.3 (38.5 to 42.1)		43.7 (42.2 to 45.1),	44.2 (42.4 to 46.0)	Personal burnout 0.003*
Cordoza, 2018	Burnout	MBI Human Services Survey EE (0-54)	21	EE: 22.95 (10.49)	EE: 18.43 (9.53)	21	EE: 22.95 (10.49)	EE: 9.71 (10.57	Change in mean intervention versus control group (95% CI) accounting for baseline: EE: 4.5 (2.1 to 6.8) vs. -.2 (-2.5 to 2.1), p<.001*
		DP (0-30)		DP: 7.43 (6.05)	DP: 5.48 (4.64)		DP: 6.14 (5.39)	DP: 6.24 (5.46)	DP: 1.8 (1.1 to 2.6) vs 0.0 (-1.3 to 1.3), p=.02*
		PA (0-48)		PA: 38.05 (6.07)	PA: 39.10 (4.39)		PA: 38.67 (6.01)	PA: 38.76 (4.94)	PA: -0.6 (-1.9 to 0.7) Vs. -0.0 (-2.0 to 2.1), p=.55

Appendix 3: Effectiveness of primary outcomes

First Author, Year	Outcome /Measurement	Scale (Scale Range)	Intervention Group(s)			Control Group			Effect (95% CI)
			N (N Post)	Pre-intervention Mean (SD)	Post-intervention Mean (SD)	N (N Post)	Pre-intervention Mean (SD)	Post-intervention Mean (SD)	
Deneckere, 2013	Burnout	Utrecht Burnout Scale: Emotional exhaustion (1-10)	17 teams 346	N/A	3.1 (unknown SD)	13 teams 235	N/A	3.8 (Unknown)	β-coefficient (SE) -0.569 (0.21), [-1.003 to -0.14]* p<.05,
		Mental detachment (1-10)			3.1 (unknown SD)			3.4 (unknown SD);	-0.319 (0.16), [-0.641 to 0.004],
		Level of competence (1-10)			7.8 (unknown SD)			7.4 (unknown SD)	0.394 (0.12) [0.147 to 0.640], p<.01.
Emani, 2020	Burnout	ProQOL Burnout Domain (0-50)	40	38.48 (13.20)	37.0 (12.72)	40	34.75 (17.16)	37.07 (12.58)	Mean difference study group: -3.73 t=.83, df=36, p=.03* Mean difference control group: .07, t=-0.9, df=38, p=.37.
	Stress	ProQOL Secondary Traumatic Stress Domain (0-50)	40	27.51 (15.50)	27.23 (16.98)	40	21.02 (13.45)	27.20 (16.73)	Mean difference study group: -6.49, t = 1.72 df = 36, p = .04* Mean difference control group: -.03, t = −0.24 df = 38, p = .81
Garland, 2012	Burnout	MBI (EE scale) (0-54)	24	N/A	30.49 (23.47)	24	N/A	25.91 (15.91)	Adjusted mean difference Shiftwork staffing (vs. standard staffing): Burnout = -6.9, *p<0.05 CI (-13.5, -0.3)(Adjusted for workload, study site, workload x study site interaction)
Gregory, 2018	Burnout	MBI	37 (Post at 3	Mean (95% CI)	Mean (95% CI)	32 (Post at 3	Mean (95% CI)	Mean (95% CI)	

Appendix 3: Effectiveness of primary outcomes

First Author, Year	Outcome /Measurement	Scale (Scale Range)	Intervention Group(s)			Control Group			Effect (95% CI)
			**N (N Post)**	**Pre-intervention Mean (SD)**	**Post-intervention Mean (SD)**	**N (N Post)**	**Pre-intervention Mean (SD)**	**Post-intervention Mean (SD)**	
			months 25, at 6 months 33)			months 35, at 6 months 37)	baseline		3 months: Decrease in EE of 6.989 units = 11% reduction (p = 0.039*)<br>Decrease in DP and SE not significant<br><br>Analyses controlled for workload<br><br>6 months: not significant for EE, DP, SE
		EE (0-54)		24.41 (20.6-28.2)	3 months: 22.36 (16.8-27.9)<br>6 months: 21.55 (17.5-25.6)		24.38 (19.1-29.6)	3 months: 24.86 (20.4-29.3)<br>6 months: 27.22 (22.7-31.8)	
		DP (0-30)		5.89 (4.2-7.6)	3 months: 5.36 (3.0-7.8)<br>6 months: 4.76 (3.3-6.3)		6.69 (4.3-9.0)	3 months: 8.29 (5.8-10.8)<br>6 months: 9.38 (6.9-11.8)	
		Self-efficacy (0-48)		42.54 (40.8-44.3)	3 months: 43.76 (41.8-45.7)<br>6 months: 42.85 (41.2-44.5)		41.81 (40.0-43.6)	3 months: 41.97 (40.5-43.4)<br>6 months: 41.00 (39.2-42.8)	
Havermans, 2018	Stress	Stress subscale of the short version of the Depression, Anxiety and Stress Scale (DASS-21) (0-21)	161	4.4 (4.0)	6 months: 4.28 (3.83); 12 months: 4.16 (3.10	143	4.0 (4.3)	6 months: 5.33 (5.05); 12 months: 4.39 (3.93)	B (overall): -0.95* (-1.81 to -0.09); B (6 months): -1.28* (-2.35 to -0.21); B (12 months): -0.51 (-1.75 to 0.74). *p<0.05, Effect size overall: .23
Jakobsen, 2017	Mental Wellbeing	Mental Health, 4-items from 36-item short form health	111 (Post: 101)	81 (13)	Change from baseline: 0.8 (-1.5 to 3.1)	89	81 (12);	Change from baseline: 0.2 (-2.3 to 2.7);	Work vs home, difference between group at follow-up: .7 (-1.7 to 3.2), p=0.56, effect size 0.06

Appendix 3: Effectiveness of primary outcomes

First Author, Year	Outcome /Measurement	Scale (Scale Range)	Intervention Group(s)			Control Group			Effect (95% CI)
			N (N Post)	Pre-intervention Mean (SD)	Post-intervention Mean (SD)	N (N Post)	Pre-intervention Mean (SD)	Post-intervention Mean (SD)	
		survey (SF-36) (0-100)							
		Vitality, 3 items from 36-item short form health survey (SF-36) (0-100)		64 (20)	Change from baseline: 5 (2 to 8)		66 (19)	Change from baseline: -2 (-3.7 to 3.1)	7 (3-10), p=0.0003*, effect size 0.36.
Kossek, 2019	Stress	PSS 4 items (4-20)	725 (Post: 420)	Baseline Total sample 9.56	6, 12, 18 months Total sample 9.34, 9.13, 9.06	799 (Post: 511)	Baseline Total sample 9.44	6, 12, 18 months Total sample 9.15, 9.03, 9.17	No significant overall effect (Total sample, over all time points)
				Childcare responsibilities 9.86	9.63, 9.56, 9.67		9.79	9.59, 9.30, 9.43	Double duty caregivers with childcare responsibilities versus no childcare responsibilities significant effects at 18 months (y=-.78, p = .002, Delta=.34)
				No childcare responsibilities 9.32	9.10, 8.76, 8.53		9.14	8.76, 8.72, 8.96	
				Elder care responsibilities 9.97	9.45, 9.19, 9.27		9.52	9.20, 9.55, 9.45	Double duty caregivers with elder care responsibilities versus no elder care responsibilities significant effect at 12 months (y=-1.08, p = .007, Delta=.35)
				No elder care responsibilities 9.36	9.28, 9.09, 8.98		9.42	9.13, 8.88, 9.10	
				Sandwich (both childcare and elder care) 10.19	9.75, 9.47, 9.73		9.57	9.39, 9.91, 9.40	Triple-duty (sandwich) caregivers versus nonsandwich caregivers significant effect at 12 months (y=-1.22, p = .024, Delta=.40)

Appendix 3: Effectiveness of primary outcomes

First Author, Year	Outcome /Measurement	Scale (Scale Range)	Intervention Group(s)			Control Group			Effect (95% CI)
			N (N Post)	Pre-intervention Mean (SD)	Post-intervention Mean (SD)	N (N Post)	Pre-intervention Mean (SD)	Post-intervention Mean (SD)	
				Nonsandwich 9.45	9.27, 9.08, 8.95		9.43	9.12, 8.90, 9.16	
	Psychological distress	K-6 Mental Health Screening Questionnaire (6-30)		Baseline Total sample 12.01	6, 12, 18 months: 11.81, 11.31, 11.12		Baseline: 11.76	6, 12, 18 months: 11.21, 11.24, 11.14	No significant overall effect (Total sample, over all time points)
				Baseline Childcare responsibilities 12.38	6, 12, 18 months: 12.20, 11.80, 11.68		Baseline: 11.93	6, 12, 18 months: 10.52, 11.30, 11.14	Double duty caregivers with childcare responsibilities versus no childcare responsibilities no significant effects over any of the time points
				Baseline No childcare responsibilities 11.71	6, 12, 18 months: 11.49, 10.88, 10.61		Baseline: 11.62	6, 12, 18 months: 10.94, 11.10, 11.12	
				Baseline Elder care responsibilities 12.53	6, 12, 18 months: 12.23, 11.20, 11.29		Baseline: 12.28	6, 12, 18 months: 11.70, 11.92, 11.61	Double-duty caregivers with elder care responsibilities versus no elder care responsibilities significant effect at 12 months (y=-1.07, p = .046, Delta=.25)
				Baseline No elder care responsibilities 11.77	6, 12, 18 months: 11.62, 11.35, 11.06		Baseline: 11.57	6, 12, 18 months: 11.07, 11.05, 11.01	
				Baseline Sandwich 12.54	6, 12, 18 months: 12.49, 11.41, 11.67		Baseline: 12.33	6, 12, 18 months: 12.03, 11.95, 11.48	Triple-duty (sandwich) caregivers versus nonsandwich caregivers no

Appendix 3: Effectiveness of primary outcomes

First Author, Year	Outcome /Measurement	Scale (Scale Range)	Intervention Group(s)			Control Group			Effect (95% CI)
			N (N Post)	Pre-intervention Mean (SD)	Post-intervention Mean (SD)	N (N Post)	Pre-intervention Mean (SD)	Post-intervention Mean (SD)	
				Baseline Nonsandwich 11.91	6, 12, 18 months: 11.69, 11.28, 11.01		Baseline: 11.68	6, 12, 18 months: 11.12, 11.10, 11.13	significant effects over any of the time points
Leiter, 2011	Burnout	MBI-GS<br>EE and CY scales<br>(EE: 0-6<br>CY: 0-6)	262 (Post: 181)	EE: 3.21 (1.57)<br>CY 1.89 (1.41)	EE: 2.76 (1.49)<br>CY: 1.36 (1.27)	911 (Post 726)	EE: 2.73 (1.42)<br>1.65 (1.26)<br>CY 1.65 (1.26)	EE: 2.65 (1.42)<br>1.65 (1.26)<br>CY 1.55 (1.28)	Overall effect 12 months: Time X CREW Intervention interaction<br>EE:<br>β -0.20, t(df) –1.53, r2 0,02%<br>CY:β -0.28, t(df) –2.47, p<.05*, r2 0,08%<br>Composite score EE and CY: 24 months: significant linear time by intervention interaction t(32)=-2.43, p=.049*
Linzer, 2015	Burnout	5-item scale, (1-5) mainly emotional exhaustion, (score of 3 or greater = indicating burnout)	82 (65 post)	% burnout 41.4% (3 or greater)	% burnout 33.8% (3 or greater)	83 (70 post)	% burnout 30.1%	% burnout 32.8%	Person-centered analysis: 21.8% fewer clinicians burned out vs. 7.1% fewer control clinicians (p<,.05). Group analyses: no significant effect
	Stress	5-point scale (1-5), high stress=4 or greater	83 (Post: 67)	30.1% high (4 or greater)	30.7% high (4 or greater)	83	27.7% high	24.2% high	Person-centered analyses: No significant differences in stress decreases (Control group: 14.3% versus 7.6% intervention group).

Appendix 3: Effectiveness of primary outcomes

First Author, Year	Outcome /Measurement	Scale (Scale Range)	Intervention Group(s)			Control Group			Effect (95% CI)
			N (N Post)	Pre-intervention Mean (SD)	Post-intervention Mean (SD)	N (N Post)	Pre-intervention Mean (SD)	Post-intervention Mean (SD)	
									Group-analyses: no significant effect
Olson, 2016	Mental Wellbeing	SF-12 mental health component composite score (1-100)	74 (Post: 64, 12 months: 63)	48.39 (SE: 1.22)	6 months, 47.95 (SE: 1.26); 12 months, 48.71 (SE: 1.29)	74 (Post: 6 months: 63, 12 months: 59)	49.29 (SE: 1.22)	6 months, 49.19 (SE: 1.28); 12 months, 49.29 (SE: 1.33)	Change from baseline to 6 months: B=-.34 (–3.59, 2.90), d=-.03<br><br>12 months: B= .31 (–3.05, 3.68), d=+.03
	Stress (secondary)	Perceived Stress Scale (not reported)	74 (Post: 64, 12 months: 63)	9.89 (SE: .62)	6 months: 9.61 (SE: .65)<br>12 months: 9.69 (SE:.66)	74 (Post: 6 months: 63, 12 months: 59)	9.84 (SE: .63)	6 months: 9.11 (SE: .66)<br>12 months: 9.42 (SE: .67)	Not significant<br>Change from baseline 6 months:<br>.45 (95%CI: -1.19 - 2.08), d= .11<br>Change from baseline 12 months: .22 (95%CI: -1.45 - 1.88), d= .05
Redhead, 2011	Burnout	MBI<br>EE (0-54)	Qualified staff: 12; Unqualified staff: 10	Qualified staff: EE=23.75 (15.46); Unqualified staff: EE=15.70 (9.13)	Qualified staff: EE=21.16 (14.08); Unqualified staff: EE=19.50 (10.42)	Qualified staff: 9; Unqualified staff: 11	Qualified staff: EE=19.44 (10.57); Unqualified staff: EE=15.73 (8.78)	Qualified staff: EE=20.11 (8.11); Unqualified staff: EE=25.0 (10.89)	Qualified staff: EE: t (19) =-0.20, p=.843, effect size d=0.09; Unqualified staff: EE: t (19) =1.18, p=.252, effect size d=0.54
		DP (0-30)		Qualified staff: DP=6.33 (6.67);	Qualified staff: DP=3.08 (2.90);		Qualified staff: DP=5.22	Qualified staff: DP=6.22 (2.48);	Qualified staff: ***DP: t (19) = 2.60, p=0.018*, effect size d=1.19;*** Unqualified staff: DP: t

Appendix 3: Effectiveness of primary outcomes

First Author, Year	Outcome /Measurement	Scale (Scale Range)	Intervention Group(s)			Control Group			Effect (95% CI)
			N (N Post)	Pre-intervention Mean (SD)	Post-intervention Mean (SD)	N (N Post)	Pre-intervention Mean (SD)	Post-intervention Mean (SD)	
				Unqualified staff: DP=4.40 (4.65)	Unqualified staff: DP=8.70 (5.89)		(3.73); Unqualified staff: DP=7.09 (5.77)	Unqualified staff: DP=9.0 (5.19)	(19) = 0.12, p=.903, effect size d=0.055
		PA (0-48)		Qualified staff: PA=33.41 (10.41); Unqualified staff: PA=31.8 (7.19)	Qualified staff: PA=35.66 (4.39); Unqualified staff: PA=33.40 (6.83)		Qualified staff: 32.44 (6.69); Unqualified staff:35.55 (6.83)	Qualified staff: PA=32.55 (7.40); Unqualified staff: PA=30.73 (7.48)	Qualified staff: PA: t (19) = -1.21, p=.243, effect size d = 0.56; Unqualified staff: PA: t (19) = -0.85, p=.405, effect size d = 0.41
Saffari, 2021	Anxiety Symptoms	State and Trait Anxiety Questionnaire (20 to 80)	Routine + Booklet: 40; Routine + Booklet + Oral: 40; Routine + Booklet + Oral + Supervision: 40	**State Anxiety:** Routine + Booklet: 55.38 (2.98); Routine + Booklet + Oral: 54.63 (2.88); Routine + Booklet + Oral + Supervision: 54.35 (2.81)	***Follow-up 1:*** **State Anxiety:** Routine + Booklet: 42.98 (3.35); Routine + Booklet + Oral: 49.28 (3.08); Routine + Booklet + Oral + Supervision: 41.95 (2.91)	40	**State Anxiety:** 54.68 (3.37)	***Follow-up 1:*** **State Anxiety:** 61.55 (3.35)	State Anxiety: between group comparison, within group comparison and interaction between time and treatments, all p < .001 *| Trait anxiety: not significant between groups: p=.233; within group p=.670; and interaction between time and treatments (overall effect): p=.769
				**Trait Anxiety**: Routine + Booklet: 54.3 (4.81); Routine	**Trait Anxiety**: Routine + Booklet: 53.13 (4.07); Routine		**Trait Anxiety:** 55.35 (4.91)	**Trait Anxiety:** 52.68 (4.69)	

Appendix 3: Effectiveness of primary outcomes

First Author, Year	Outcome /Measurement	Scale (Scale Range)	Intervention Group(s)			Control Group			Effect (95% CI)
			N (N Post)	Pre-intervention Mean (SD)	Post-intervention Mean (SD)	N (N Post)	Pre-intervention Mean (SD)	Post-intervention Mean (SD)	
				+ Booklet + Oral: 55.23 (4.16); Routine + Booklet + Oral + Supervision: 55.38 (4.48)	+ Booklet + Oral: 54.65 (3.87); Routine + Booklet + Oral + Supervision: 53.78 (4.25)				
					Follow-up 2: **State Anxiety**: Routine + Booklet: 30.35 (3.79); Routine + Booklet + Oral: 37.0 (3.57); Routine + Booklet + Oral + Supervision: 29.63 (3.51)			***Follow-up 2:*** **State Anxiety:** 66.03 (2.42) |	
					**Trait Anxiety**: Routine + Booklet: 52.3 (4.64); Routine + Booklet + Oral: 52.33 (4.40); Routine + Booklet + Oral +			**Trait Anxiety:** 53.85 (4.02)	

Appendix 3: Effectiveness of primary outcomes

First Author, Year	Outcome /Measurement	Scale (Scale Range)	Intervention Group(s)			Control Group			Effect (95% CI)
			N (N Post)	Pre-intervention Mean (SD)	Post-intervention Mean (SD)	N (N Post)	Pre-intervention Mean (SD)	Post-intervention Mean (SD)	
					Supervision: 54.1 (3.47)				
					Follow-up 3: **State Anxiety:** Routine + Booklet: 25.3 (3.63); Routine + Booklet + Oral: 26.08 (3.68); Routine + Booklet + Oral + Supervision: 22.93 (2.72) | **Trait Anxiety:** Routine + Booklet: 56.3 (4.64); Routine + Booklet + Oral: 56.33 (4.40); Routine + Booklet + Oral + Supervision: 58.1 (3.47)			***Follow-up 3:*** **State Anxiety:** 68.18 (1.57) | **Trait Anxiety:** 57.85 (4.03)	
	Stress	Perceived Stress Questionnaire (PSQ-14) (0 to 70)		Routine + Booklet: 34.78 (2.91); Routine	***Follow-up 1:*** Routine + Booklet: 29.35	40	36.05 (3.11)	***Follow-up 1:*** ***48.37 (3.69***)	Between group comparison, within group comparison and

Appendix 3: Effectiveness of primary outcomes

First Author, Year	Outcome /Measurement	Scale (Scale Range)	Intervention Group(s)			Control Group			Effect (95% CI)
			N (N Post)	Pre-intervention Mean (SD)	Post-intervention Mean (SD)	N (N Post)	Pre-intervention Mean (SD)	Post-intervention Mean (SD)	
				+ Booklet + Oral: 35.03 (2.99); Routine + Booklet + Oral + Supervision: 34.45 (3.19)	(4.38); Routine + Booklet + Oral: 31.87 (3.24); Routine + Booklet + Oral + Supervision: 29.6 (3.46)				interaction between time and treatments, all p < .001*
					Follow-up 2: Routine + Booklet: 27.6 (4.56); Routine + Booklet + Oral: 28.8 (3.42); Routine + Booklet + Oral + Supervision: 26.65 (3.85)			***Follow-up 2:*** 50.65 (3.75)	
					Follow-up 3: Routine + Booklet: 24.53 (4.44); Routine + Booklet + Oral: 25.85 (3.86); Routine + Booklet + Oral +			***Follow-up 3:*** 61.1 (4.06)	

Appendix 3: Effectiveness of primary outcomes

First Author, Year	Outcome /Measurement	Scale (Scale Range)	Intervention Group(s)			Control Group			Effect (95% CI)
			N (N Post)	Pre-intervention Mean (SD)	Post-intervention Mean (SD)	N (N Post)	Pre-intervention Mean (SD)	Post-intervention Mean (SD)	
					Supervision: 21.73 (4.35)				
Stansfeld, 2015	Mental Wellbeing	Warwick Edinburgh Mental Wellbeing Scale (WEMWBS) (14-70)	Employees 225	51.0 (8.3)	49.9 (8.3)	59	50.4 (8.0)	49.0 (8.5)	Mean difference adjusted for baseline and clustering: .5 (-3.2 to 4.2)
	Stress	General Health Questionnaire-12 (GHQ-12) (0-12)	Employees 225	2.8 (3.5)	2.9 (3.5)	59	3.2 (3.4)	2.9 (3.7)	Mean difference adjusted for baseline and clustering GHQ-12: .2 (-2.0 to 2.5)
Tran, 2010	Stress	Stress in General (SIG) with two subscales: pressure at work (0-14) and work-related threat (0-16)	74 (Post: 39)	Pressure: 12.38 (3.96); Threat: 8.51 (5.61)	Pressure: 12.55 (3.43); Threat: 8.78 (4.77)	51 (Post: 14)	Pressure: 11.98 (4.18); Threat: 7.87 (5.84)	Pressure: 11.62 (4.46); Threat: 8.00 (5.94)	Within and between group differences not significant for pressure and threat
Uchiyama, 2013	Depressive Symptoms	Center for Epidemiologic Studies Depression Scale (CES-D) (0 to 60)	133	16.1 (9.4)	15.1 (9.7)	154	15.8 (9.6)	15.2 (8.9)	No significant pre-post changes in study and control group<br>No significant overall effect: F = 0.132, p=.717
West, 2014	Depressive Symptoms	Positive depression screening: Patient Health Questionnaire (PHQ-2) (0-2, cut-off =1)	37	N (%)<br>11 (29.7%)	Change %<br>3 months= +2.7%; 12 months= -6.2%	37	N (%)<br>11 (31.4%)	Change (%)<br>3 months = +1.0%;<br>12 months = -4.1	3 months post intervention adjusted for baseline: P=.60<br>12 months post intervention adjusted for baseline: P=.62

Appendix 3: Effectiveness of primary outcomes

First Author, Year	Outcome /Measurement	Scale (Scale Range)	Intervention Group(s)			Control Group			Effect (95% CI)
			N (N Post)	Pre-intervention Mean (SD)	Post-intervention Mean (SD)	N (N Post)	Pre-intervention Mean (SD)	Post-intervention Mean (SD)	
	Burnout	MBI<br>High EE (>= 14)<br>High DE (>=14)	37	N (%)<br>High DE = 9(24.3%); high EE = 17(45.9%); overall high burnout = 20 (54.1%)	N (%)<br>3 months post int (changes from baseline): High DE = -15.5; high EE = -16.5; overall high burnout = -24.7; 12 months post int DE = -9.6; EE = -19.4; overall burnout = -21.7	37	N (%)<br>High DE = 9(25.4%); high EE = 12(34.3%); overall high burnout = 15 (42.9%)	N (%<br>3 months post int (changes from baseline): High DE = +0.8; high EE = -7.8; overall high burnout = -7.6; @12 months post int: High DE = -1.5; High EE = -16.1; overall high burnout = -15.6	All analyses adjusted for baseline levels<br>High DE: 3 months: p=.004*, 12 months: p=.02*<br>High EE: 3 months: p=.54; 12 months: p=.69<br>Overall high burnout: 3 months: p= 14,<br>12 months: p=.22
	Stress	Perceived Stress Scale (10 items) (0-40)	37	18.0 (5.6)	Change in mean: 3 months = -3.2; 12 months = -2.6	37	16.2 (6.2)	Change in mean: 3 months = -2.3; 12 months = -0.8	3 months post intervention adjusted for baseline, p=.83<br>12 months post intervention adjusted for baseline, p=.58
White, 2010	Mental Wellbeing	SF-8<br>(Mental Component score)	54<br>Complete data at 2 timepoints	Median (range)<br>51.5 (19-60)	Median (range)<br>51.0 (18-63)	59 (42)<br>12 Complete data for 2 time points	Median (range)<br>50.3 (25-69)	Median range)<br>49.8 (26-59)	No significant changes in either group.<br>Intervention group: Z –1.569, p=.117,<br>Control group: Z=-.706, p=.480

Appendix 3: Effectiveness of primary outcomes

First Author, Year	Outcome /Measurement	Scale (Scale Range)	Intervention Group(s)			Control Group			Effect (95% CI)
			N<br>(N Post)	Pre-intervention Mean (SD)	Post-intervention Mean (SD)	N<br>(N Post)	Pre-intervention Mean (SD)	Post-intervention Mean (SD)	
									No between-group comparisons reported
	Depressive Symptoms	GHQ 28 subscale	54<br>Complete data at 2 timepoints	Median (range)<br>0 (0-8)	Median (range)<br>0 (0-13)	59 (42)<br>12 Complete data for 2 timepoints	Median (range)<br>0 (0-9)	Median (range)<br>0 (0-7)	No significant changes in either group.<br>Intervention group: Z –1.346, p=.17,<br>Control group: .000, p=1.00<br>No between-group comparisons reported
	Anxiety Symptoms	GHQ 28 subscale	54<br>Complete data at 2 time points	Median (range)<br>3.5 (0-13)	Median (range)<br>4.0 (0-18)	59 (42)<br>12 Complete data for 2 timepoints	Median (range)<br>4 (0-16)	Median (range)<br>5.5 (0-18)	No significant changes in either group.<br>Intervention group: -1.317, p=.188<br>Control group: Z=-.358, p=.720<br>No between-group comparisons reported
	Burnout	MBI - EE, PA, DE and Total score	54<br>Complete data at 2 timepoints	Median (range)<br>EE = 15.0 (0-52); DE = 4.0 (0-21); PA = 37.0 (12-47)	Median (range)<br>EE=15.5 (0-44);<br>DE= 4.0 (0-20);<br>PA = 37.0 (18-48)	59 (42)<br>12 Complete data for 2 timepoints	Median (range)<br>EE = 16 (1-53); DE = 4.0 (0-27); PA = 36.0 (8-48)	Median (range)<br>EE=19.0 (0-45);<br>DE=6.0 (0-20);<br>PA: 35.0 (10-46)	No significant changes in either group.<br>Intervention group:<br>EE: Z=-.186, p=.853<br>DE: Z=.138, p=.890<br>PA: Z=-.859, p=.390<br>Control group:<br>EE: Z=-.756, p=.449<br>DE: Z=-.256, p=.798<br>PA: Z=-1.292, p=.196<br>No between group comparisons reported

Appendix 3: Effectiveness of primary outcomes

First Author, Year	Outcome /Measurement	Scale (Scale Range)	Intervention Group(s) N (N Post)	Intervention Group(s) Pre-intervention Mean (SD)	Intervention Group(s) Post-intervention Mean (SD)	Control Group N (N Post)	Control Group Pre-intervention Mean (SD)	Control Group Post-intervention Mean (SD)	Effect (95% CI)
Van Woerkom, 2021	Mental Wellbeing	Dutch version of the Everyday Feeling Questionnaire (EFQ) with adaptation (reference to momentary wellbeing). (1-5)	18<br>20<br>29	**Intensive care** ward INT 1 nap facility = **3.86** (n=18); **Paeds** INT 2 glasses = **3.70** (n=20); **Emergency** INT 1 & 2 = **3.92** (n=29)		28	**Medium care 4.06** (n=16); **Orthopedics 3.48** (n=12)		Access to facilities (experimental conditions): B (SE), p<br>Nap facility: .05, (.10), p=.62<br>Glasses: .08 (.09), p=.38<br>Nap facility + glasses: .38 (.09), p=<.001*<br><br>Use of facilities:<br>Glasses and napping facility: (B = .04, ns), Use glasses and slept in napping facility (B = .11, ns) and glasses and time spent sleeping in napping facility. M3: (**B = .01, p < .05**). Use of light therapy glasses was in all models positively associated with psychological well-being T2 (respectively **B = .17, p < .05; B = .17, p < .05; and B = .16, p < .05).**

Note: MBI = Maslach Burnout Inventory, MBI-GS = Maslach Burnout Inventory General Survey, EE=Emotional Exhaustion, DP=Depersonalisation, CY= Cynism, SD=Standard deviation, SE=Standard error, CI=Confidence interval
