# Supplementary material for: The effects of different types of organisational workplace mental health interventions on mental health and wellbeing in healthcare workers: a systematic review
Source: Int Arch Occup Environ Health. 2024 May 2;97(5):485–522. doi: 10.1007/s00420-024-02065-z (PMC11130054; doi:10.1007/s00420-024-02065-z)
Supplement: Supplementary file 4 — Supplementary file1 (PDF 1114 KB) Appendix 4: Effectiveness of secondary outcomes [file 420_2024_2065_MOESM4_ESM.pdf]

Appendix 4: Effectiveness of secondary outcomes

			Intervention group			Control group			
**First Author, Year**	**Outcome/Measurement**	**Scale Range**	**N (N Post)**	**Pre-Intervention Mean (SD)**	**Post-Intervention Mean (SD)**	**N (N Post)**	**Pre-Intervention Mean (SD)**	**Post-Intervention Mean (SD)**	**Measure of effect (95% CI)**
Ali, 2011	Work-Home Life Imbalance	2-10			5.6 (1.81)			6.72 (2.24)	Adjusted difference: 1.02 (0.10 to 1.93), p=.029*
Barcons, 2019	No Secondary Outcomes								
Bourbonnais, 2011	Psychological demands (JCQ)	(6-21)	492 (248)	M0 means (95% CI), SD<br><br>Psychological demands 12.5 (12.2 to 12.8), 4.0	Means (95% CI) at M2 adjusted for M0<br>Psychological demands 11.9 (11.5 to 12.3)	618 (240)	M0 means (95% CI), SD<br><br>Psychological demands 13.3 (13.0 to 13.7), 3.6	Means (95% CI) at M2 adjusted for M0<br>Psychological demands 12.6 (12.2 to 13.0)	Means at M2 adjusted for M0<br><br>Psychological demands P=0.008*
	Effort-Reward Imbalance (ERI questionnaire for rewards, demands from JCQ)	(0.24-4)		Effort-reward imbalance 1.1 (1.1 to 1.1), 0.3	Effort-reward imbalance 1.01 (1.0 to 1.1)		Effort-reward imbalance 1.2 (1.2 to 1.2), 0.3	Effort-reward imbalance 1.1 (1.1 to 1.2)	Effort-reward imbalance p<0.001*
	Quality of Work	(5-20)		Quality of work 12.2 (11.9 to 12.4), 2.2	Quality of work 12.7 (12.4 to 12.9)		Quality of work 12.2 (12.0 to 12.4) 2.2	Quality of work 12.1 (11.9 to 12.3)	Quality of work P=0.002*
	Emotional demands	(7-28)		Emotional demands	Emotional demands		Emotional demands	Emotional demands	Emotional demands

Appendix 4: Effectiveness of secondary outcomes

			Intervention group			Control group			
**First Author, Year**	**Outcome/Measurement**	**Scale Range**	**N (N Post)**	**Pre-Intervention Mean (SD)**	**Post-Intervention Mean (SD)**	**N (N Post)**	**Pre-Intervention Mean (SD)**	**Post-Intervention Mean (SD)**	**Measure of effect (95% CI)**
				17.2 (16.8 to 17.6)	16.5 (16.0 to 17.0)		17.2 (16.8 to 17.6) 4.1	16.3 (15.8 to 16.8)	P=0.525
Cordoza, 2018	No Secondary Outcomes								
Deneckere, 2013	No Secondary Outcomes								
Emani, 2020	No Secondary Outcomes								
Garland, 2012	Work-Home Imbalance 8 item scale (Kopelman et al 1983)	5-40			Intervention vs. Control: -10.1, p<.05				Shiftwork staffing (vs. standard staffing): Adjusted (workload, site, interaction workload x site) mean difference = -10.1 (*p<0.05): CI(-19.5, -0.7)
	Job Overload 3 item scale (Bacharach et al. 1990)	3-12			Intervention vs. Control: -28.4, p<.05				Shiftwork staffing (vs. standard staffing): adjusted (workload, site, interaction workload x site) mean difference

Appendix 4: Effectiveness of secondary outcomes

			Intervention group			Control group			
**First Author, Year**	**Outcome/Measurement**	**Scale Range**	**N (N Post)**	**Pre-Intervention Mean (SD)**	**Post-Intervention Mean (SD)**	**N (N Post)**	**Pre-Intervention Mean (SD)**	**Post-Intervention Mean (SD)**	**Measure of effect (95% CI)**
									= -28.4 (*p<0.01): CI(-37.7, -19.1) Negative values indicate improvement for the intervention group
	Role Uncertainty 5 item scale (Peterson et al. 1995)	5-25			Intervention vs. Control: 9.5, p<.05				Shiftwork staffing (vs. standard staffing): adjusted (workload, site, interaction workload x site) mean difference = 9.5 (*p<0.05): CI(2.2, 16.7) Negative values indicate improvement for the intervention group, positive

Appendix 4: Effectiveness of secondary outcomes

			Intervention group			Control group			
**First Author, Year**	**Outcome/Measurement**	**Scale Range**	**N (N Post)**	**Pre-Intervention Mean (SD)**	**Post-Intervention Mean (SD)**	**N (N Post)**	**Pre-Intervention Mean (SD)**	**Post-Intervention Mean (SD)**	**Measure of effect (95% CI)**
									outcomes the reverse
Gregory, 2018	Workload Areas of Work life Scale (AWS) (higher = more congruence between actual and expected workload)	1-5	37 (Post at 3 months 25, at 6 months 33)	Baseline 2.50 (2.2-2.8)	3 months 2.99 (2.6-3.4) 6 months 2.62 (2.3-2.9)	32 (Post at 3 months 35, at 6 months 37)	Baseline 2.46 (2.1-2.8)	3 months 2.38 (2.1-2.7) 6 months 2.32 (2.0-2.6)	Improvement in workload of 0.61 units (p = 0.037) (only after 3 months)
Havermans, 2018	Psycho-logical Demands, 5 item subscale from JCQ	Range 5-20	161 at baseline (111 used in analysis)	14.75 (2.29)	6 months: 14.62 (2.43); 12 months: 14.32 (2.12)	143 at baseline (99 used in analysis)	14.21 (2.39)	6 months: 14.24 (2.31); 12 months: 14.45 (2.27)	B (overall): -.25 (-0.74 to .25); B (6 months): 0.01 (-.57 to 0.60); B (12 months): -.63 (-1.31 to 0.04). (adjusted for age and working night shifts)
	Social Support, 4 items subscale for co-worker	Range 4-16		12.27 (1.65)	6 months: 12.56 (1.72); 12 months: 12.55 (1.74)		12.68 (1.84)	6 months: 12.47 (1.70); 12 months: 12.99 (1.75)	B (overall): 0.02 (-0.58 to 0.63); B (6 months): 0.20 (-0.45 to

Appendix 4: Effectiveness of secondary outcomes

			Intervention group			Control group			
First Author, Year	Outcome/Measurement	Scale Range	N (N Post)	Pre-Intervention Mean (SD)	Post-Intervention Mean (SD)	N (N Post)	Pre-Intervention Mean (SD)	Post-Intervention Mean (SD)	Measure of effect (95% CI)
	support from JCQ								0.85); B (12 months): -0.21 (-0.90 to 0.48). (adjusted for age and working night shifts)
	Social Support, 4 item subscale for supervisor support from JCQ	Range 4-16		10.54 (2.07)	6 months: 10.98 (2.06); 12 months: 11.36 (2.12)		10.43 (2.39)	6 months: 11.52 (1.87); 12 months: 11.37 (2.07)	B (overall): -0.36 (-0.80 to 0.08); B (6 months): -0.50 (-1.04 to 0.04); B (12 months): -0.15 (-0.78 to 0.48). adjusted for age and working night shifts)
	Autonomy, 3-item subscale from JCQ	Range 3-12		7.65 (1.00)	6 months: 7.73 (0.93); 12 months: 7.88 (0.76)		7.68 (1.11)	6 months: 7.69 (0.85); 12 months: 7.86 (0.78)	B (overall): 0.02 (-0.18 to 0.22); B (6 months): 0.03 (-0.21 to 0.27); B (12 months): 0.004 (-0.27 to 0.28). (adjusted for age and working night shifts)

Appendix 4: Effectiveness of secondary outcomes

			Intervention group			Control group			
**First Author, Year**	**Outcome/Measurement**	**Scale Range**	**N (N Post)**	**Pre-Intervention Mean (SD)**	**Post-Intervention Mean (SD)**	**N (N Post)**	**Pre-Intervention Mean (SD)**	**Post-Intervention Mean (SD)**	**Measure of effect (95% CI)**
Jakobsen, 2017	Psychosocial Working Conditions Single questions from COPSOC II<br><br>Work pace	Range 0-100 (5-point scale converted to a score of 0 to 100 (higher score is worse)).	111 (Post: 101)	Work pace 69 (17)	Work pace (change from baseline): 5.1 (2.2 to 7.9, 95%CI)	89	Work pace 71 (17)	Work pace (change from baseline): -0.10 (-3.2 to 3.1, 95%CI)	Work vs home, difference between group at follow-up: Work pace 4.6 (1.5 to 7.7, 95%CI), p=0.0042, effect size 0.27
	Emotional Demands			Emotional demands 46 (19)	Emotional demands (change from baseline): 3 (-0.6 to 6.5, 95%CI)		Emotional demands 46 (18)	Emotional demands (change from baseline): 1.4 (-2.4 to 5.3, 95%CI)	Emotional demands: 1.6 (-2.2 to 5.5, 95%CI), p=0.4054, effect size 0.09
	Influence at Work			Influence at work 38 (20)	Influence (change from baseline): -0.5 (-3.7 to 2.8, 95%CI)		Influence at work 39 (20)	Influence (change from baseline): -1.8 (-5.2 to 1.7, 95%CI)	Influence 1 (-2.4 to 4.5, 95%CI), p=0.5588, effect size 0.05
	Sense of Community at Work			Sense of community at work 15 (19)	Sense of community (change from baseline): -2.2 (-5.1 to 0.8, 95%CI)		Sense of community at work 15 (15)	Sense of community (change from baseline): -	Sense of community -1.2 (-4.4 to 1.9, 95%CI),

Appendix 4: Effectiveness of secondary outcomes

			Intervention group			Control group			
**First Author, Year**	**Outcome/Measurement**	**Scale Range**	**N (N Post)**	**Pre-Intervention Mean (SD)**	**Post-Intervention Mean (SD)**	**N (N Post)**	**Pre-Intervention Mean (SD)**	**Post-Intervention Mean (SD)**	**Measure of effect (95% CI)**
								0.8 (-4 to 2.5, 95%CI)	p=0.4432, effect size 0.07
	Social Support from Supervisor			Social support from supervisor 24 (24)	Social support from supervisor (change from baseline): 2.5 (-1.6 to 6.5, 95%CI)		Social support from supervisor 18 (21)	Social support from supervisor (change from baseline): 3.5 (-0.9 to 8, 95%CI)	Social support from supervisor -0.4 (-4.8 to 4, 95%CI), p=0.871, effect size 0.02
Kossek, 2019	No secondary outcomes								
Leiter, 2011	Self-reported absences: Number of occasions due to illness or disability		262 (Post: 181)	Monthly mean (SD) .88 (2.31)	Monthly mean (SD) .54 (1.07)	911 (Post 726)	Monthly mean (SD) .86 (2.04)	Monthly mean (SD) .83 (2.00)	Time X Intervention interaction effect at 12 months<br><br>β -.63, t(df) – 4.32* (1554) *p <.05<br>Time X Intervention effect at 24 months not significant:

Appendix 4: Effectiveness of secondary outcomes

			Intervention group			Control group			
**First Author, Year**	**Outcome/Measurement**	**Scale Range**	**N (N Post)**	**Pre-Intervention Mean (SD)**	**Post-Intervention Mean (SD)**	**N (N Post)**	**Pre-Intervention Mean (SD)**	**Post-Intervention Mean (SD)**	**Measure of effect (95% CI)**
									t(1905) = -.40, p=.69
Linzer, 2015	No Secondary Outcomes								
Olson, 2016	No Secondary Outcomes								
Redhead, 2011	No Secondary Outcomes								
Saffari, 2021	No Secondary Outcomes								
Stansfeld, 2015	HR data on sickness absence days excluding persons with absences greater than 21 days at baseline	Range 0-21	294	1.2 (3.2)	1.6 (3.7)	66	0.9 (2.0)	1.0 (1.7)	Intervention effect adjusted for baseline and clustering: 0.6 (-1.4 to 2.6), not significant
	Self-reported Sickness Absence: short term (<7 days) and medium-term (7–21 days) excluding persons with absences	Range 0-21	198	1.0 (3.0)	1.3 (3.4)	51	1.2 (3.5)	1.3 (3.8)	Intervention effect adjusted for baseline and clustering: 0.1 (-2.2 to 2.4), not significant

Appendix 4: Effectiveness of secondary outcomes

			Intervention group			Control group			
**First Author, Year**	**Outcome/Measurement**	**Scale Range**	**N (N Post)**	**Pre-Intervention Mean (SD)**	**Post-Intervention Mean (SD)**	**N (N Post)**	**Pre-Intervention Mean (SD)**	**Post-Intervention Mean (SD)**	**Measure of effect (95% CI)**
	great than 21 days at baseline								
	Supervisor Relationship JCQ abbreviated version		224	72 (20)	71 (21)	59	74 (21)	75 (19)	Intervention effect adjusted for baseline and clustering: -3.3 (-14.1 to 7.5), not significant
	Supervisor Support JCQ abbreviated version		228	80 (23)	80 (24)	59	87 (23)	86 (21)	Intervention effect adjusted for baseline and clustering: -3.2 (-19.2 to 12.9), not significant
Tran, 2010	Role Conflict (Rizzo et al 1970)	1-7	74 (Post: 39)	3.48 (1.14)	3.48 (1.21)	51 (Post: 14)	3.28 (1.07)	3.51 (1.10)	Mean difference (follow-up-baseline) Intervention .06 (1.26) Control -.15 (.63), not significant
	Role Ambiguity Scale	1-7	74 (Post: 39)	2.64 (0.91)	2.80 (0.95)	51 (Post: 14)	2.72 (1.19)	3.11 (1.42)	Mean difference (follow-up-baseline)

Appendix 4: Effectiveness of secondary outcomes

			Intervention group			Control group			
**First Author, Year**	**Outcome/Measurement**	**Scale Range**	**N (N Post)**	**Pre-Intervention Mean (SD)**	**Post-Intervention Mean (SD)**	**N (N Post)**	**Pre-Intervention Mean (SD)**	**Post-Intervention Mean (SD)**	**Measure of effect (95% CI)**
	(Rizzo et al 1970)								Intervention 0.23 (1.32) Control -0.18 (.83), not significant
Uchiyama, 2013	Job Content Questionnaire (JCQ)	Job demands (12-48)	147	35.1 (4.5)	35.1 (4.8)	163	36.3 (5.3)	36.5 (5.2)	F = 0.160, p=.690
		Job control (24-96)	146	67.6 (8.2)	68.9 (8.0)	162	67.9 (7.8)	67.6 (7.4)	F = 3.840, p=.051
		Supervisor support (4-16)	147	11.4 (2.5)	11.5 (2.3)	163	11.2 (2.5)	11.2 (2.3)	F = 0.015, p=.902
		Co-worker support (4-16)	145	11.9 (1.7)	12.4 (1.8)	163	12.0 (2.0)	11.9 (1.8)	F = 7.120, p=.008*
	Effort-Reward-Imbalance Questionnaire (ERIQ)	Effort (3-12)	142	8.7 (1.7)	9.0 (1.8)	165	8.9 (1.9)	9.2 (1.8)	F = 0.033, p=.855
		Reward (7-28)	138	18.0 (2.8)	18.2 (2.8)	163	17.9 (2.7)	18.0 (2.8)	F = 0.046, p=.831
	Quality Work Competence	Realistic and well-defined	149	58.3 (20.4)	59.7 (18.0)	163	60.0 (16.5)	55.1 (17.9)	Interaction time X group:

Appendix 4: Effectiveness of secondary outcomes

			Intervention group			Control group			
**First Author, Year**	**Outcome/Measurement**	**Scale Range**	**N (N Post)**	**Pre-Intervention Mean (SD)**	**Post-Intervention Mean (SD)**	**N (N Post)**	**Pre-Intervention Mean (SD)**	**Post-Intervention Mean (SD)**	**Measure of effect (95% CI)**
	Questionnaire (QWC)	Goals (0-100							F = 8.792, p=.003*
		Efficiency, planning (0-100)	148	58.1 (18.1)	60.7 (17.1)	164	56.0 (16.3)	55.6 (16.4)	Interaction time x group F=2.560, p=.111
		Participatory management (0-100)	148	61.4 (21.9)	65.1 (20.8)	164	59.3 (18.3)	61.2 (18.7)	Interaction time x group F=.748, p=.388
		Competence development (0-100)	147	64.4 (18.6)	64.9 (17.3)	165	62.7 (18.8)	60.6 (18.3)	Interaction time x group F = .852, p=.375
		Work Climate (0-100)	148	60.3 (15.7)	60.5 (16.6)	166	58.8 (18.2)	57.7 (15.3)	Interaction time x group F=608, p=.436
		Leadership (0-100)	144	57.5 (19.4)	58.2 (16.6)	161	54.2 (18.4)	54.5 (16.5)	Interaction time x group F=.142, p=.706
		Feedback (0-100)	147	62.3 (22.3)	61.6 (20.2)	166	59.8 (17.7)	59.2 (17.3)	Interaction time x group F=.092, p=.762
West, 2014	No secondary outcomes								

Appendix 4: Effectiveness of secondary outcomes

			Intervention group			Control group			
**First Author, Year**	**Outcome/Measurement**	**Scale Range**	**N (N Post)**	**Pre-Intervention Mean (SD)**	**Post-Intervention Mean (SD)**	**N (N Post)**	**Pre-Intervention Mean (SD)**	**Post-Intervention Mean (SD)**	**Measure of effect (95% CI)**
White, 2010	No Secondary Outcomes								
Van Woerkom, 2021	No Secondary Outcomes								

Notes:

JCQ Job Content Questionnaire

COPSOQ Copenhagen Psychosocial Questionnaire
